# Supplementary material for: Structural Basis for Selective Interaction between the ESCRT Regulator HD-PTP and UBAP1
Source: Structure. 2016 Dec 6;24(12):2115–26. doi: 10.1016/j.str.2016.10.006 (PMC5145805; doi:10.1016/j.str.2016.10.006)
Supplement: Document S1. Figures S1–S5 [file mmc1.pdf]

Structure, Volume *24*

Supplemental Information

Structural Basis for Selective Interaction between the ESCRT Regulator HD-PTP and UBAP1

Deepankar Gahloth, Colin Levy, Graham Heaven, Flavia Stefani, Lydia Wunderley, Paul Mould, Matthew J. Cliff, Jordi Bella, Alistair J. Fielding, Philip Woodman, and Lydia Tabernero

Supplementary Information

Supplementary Figures: S1, S2, S3, S4 and S5

Supplementary Movie 1

Supplementary Figure S1

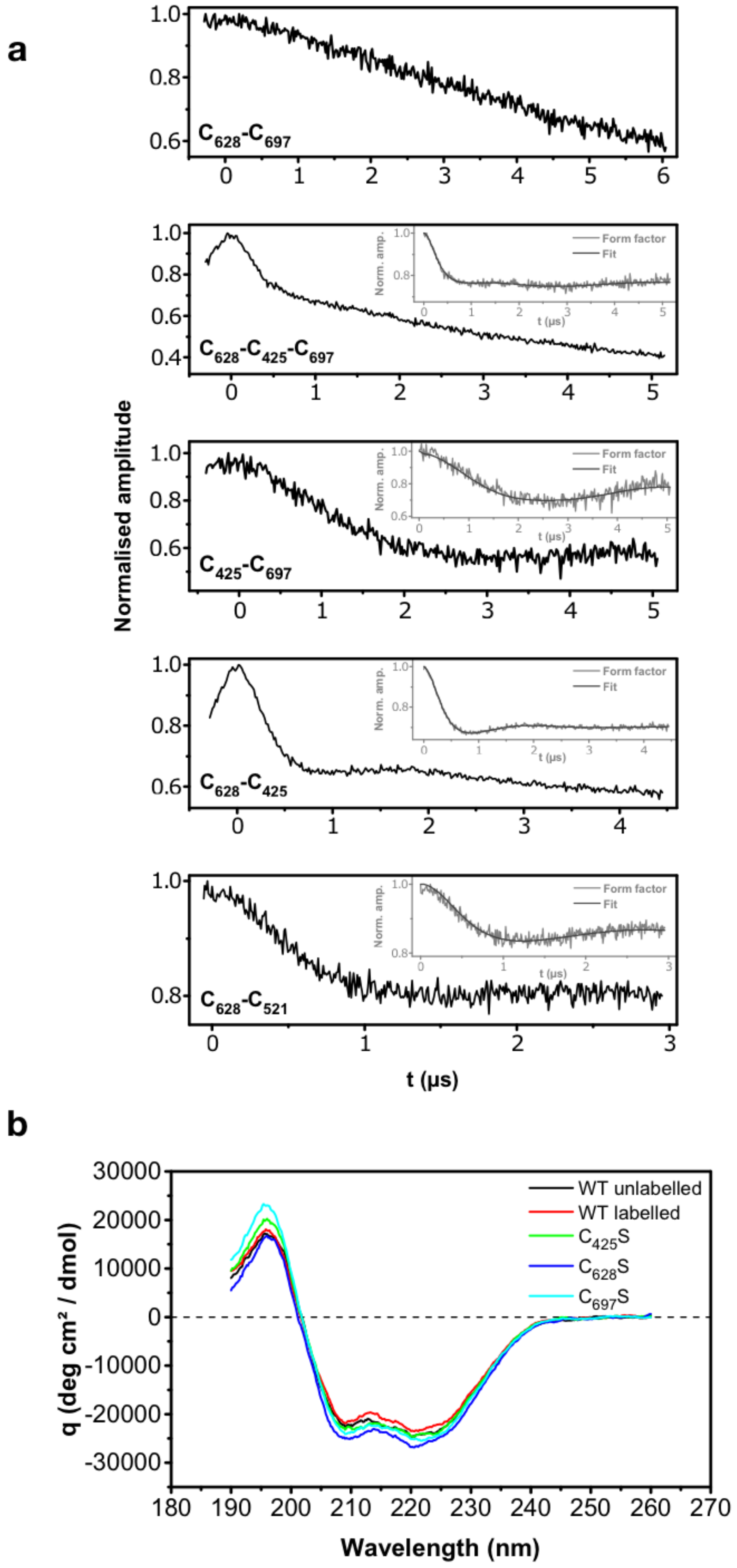

Figure S1. (Related to Figure 2) DEER spectroscopy of HD-PTP$_{CC}$ and Circular Dichrosim analysis of MTSL-labelled HD-PTP$_{CC}$ and mutants used in the EPR studies.

(a) DEER traces of the triply MTSL-labelled HD-PTP$_{CC}$ and doubly MTSL-labelled mutants, as indicated inside each frame. Inset graphs show form factor fits after exponential background correction. Modulation is apparent in all triply and doubly labelled proteins except for the C$_{425}$S mutant (top panel) where the distance between spins is larger than the detectable limits within this window size. **(b)** Circular dichroism of HD-PTP$_{CC}$ (unlabelled and triply labelled at C$_{425}$, C$_{628}$ and C$_{697}$) and mutants C$_{425}$S (labelled at C$_{628}$ and C$_{697}$), C$_{628}$S (labelled at C$_{425}$ and C$_{697}$) and C$_{697}$S (labelled at C$_{425}$ and C$_{628}$). Samples for circular dichroism were adjusted to 0.1 mg/mL and buffer exchanged into phosphate buffer (100 mM potassium phosphate, 100 mM potassium fluoride, pH 7.4). Spectra were recorded on a Jasco J-180 spectropolarimeter between 190 and 260 nm using cuvettes of 0.5 mm path length. A data pitch of 0.2 nm was used with a response time of 8 seconds per point. Circular dichroism mdeg units were converted to mean residue ellipticity. No significant changes are seen upon labelling or mutagenesis of HD-PTP$_{CC}$, thus confirming that there are no alterations to the secondary structure.

Supplementary Figure S2

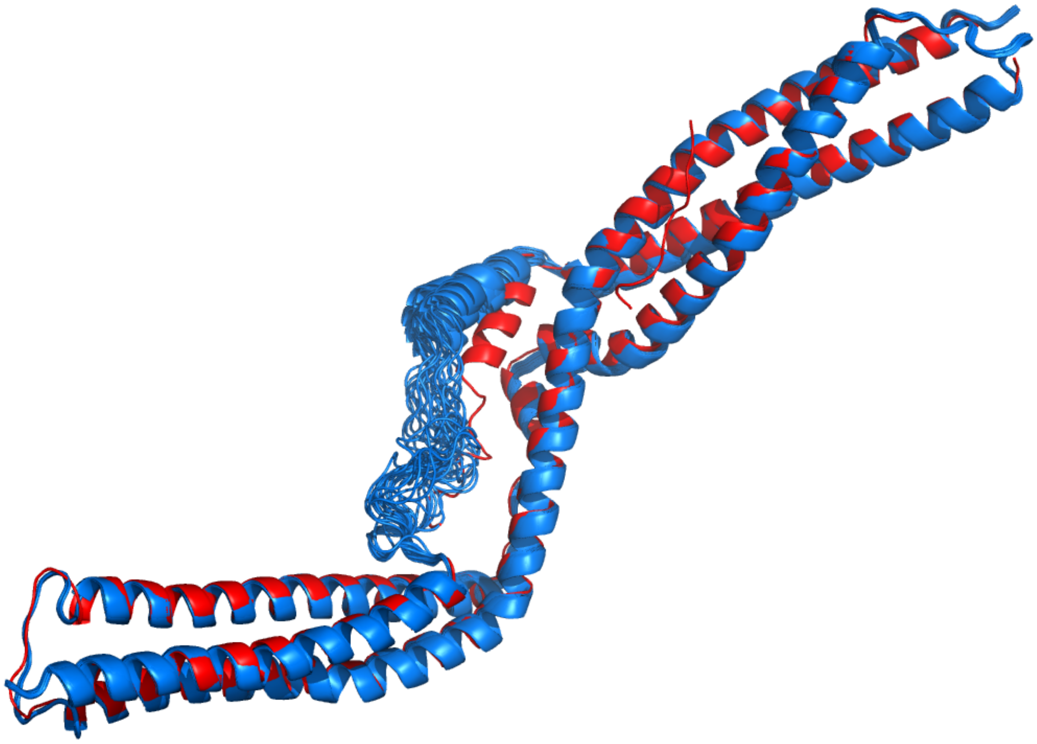

Figure S2. (Related to Figure 2) Torsion-angle molecular dynamics simulations using DEER distance constraints.

Ribbon diagram of the $HDPTP_{CC}$ crystal structure (red) superimposed to 25 trajectory model structures at 1 ns intervals (blue) showing the flexibility of the loops that connect helix H2 with the rest of the CC domain. Several orientations of helix H2 resulting from the molecular dynamics simulations are compatible with the interspin distances estimated form the DEER experiments, without major global conformational changes of the CC domain structure.

Supplementary Figure S3

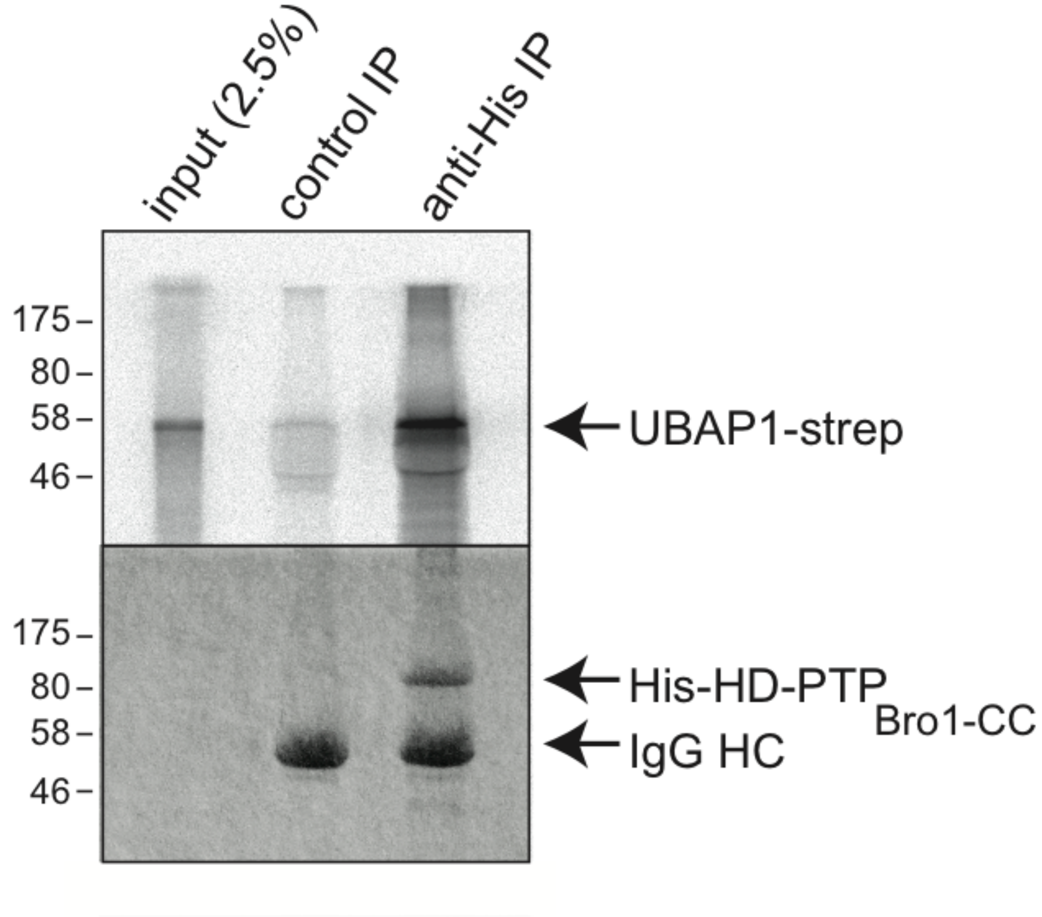

Figure S3. (Related to Figure 3) Co-immunoprecipitation of UBAP1 with HD-PTP.

UBAP1-strep was translated *in vitro* and incubated with or without His_6-HD-$PTP_{Bro1\text{-}CC}$. Samples were immunoprecipitated with anti-His antobodies. Top panel: phosphorimage. Bottom panel: Coomassie stained SDS-PAGE.

Supplementary Figure S4

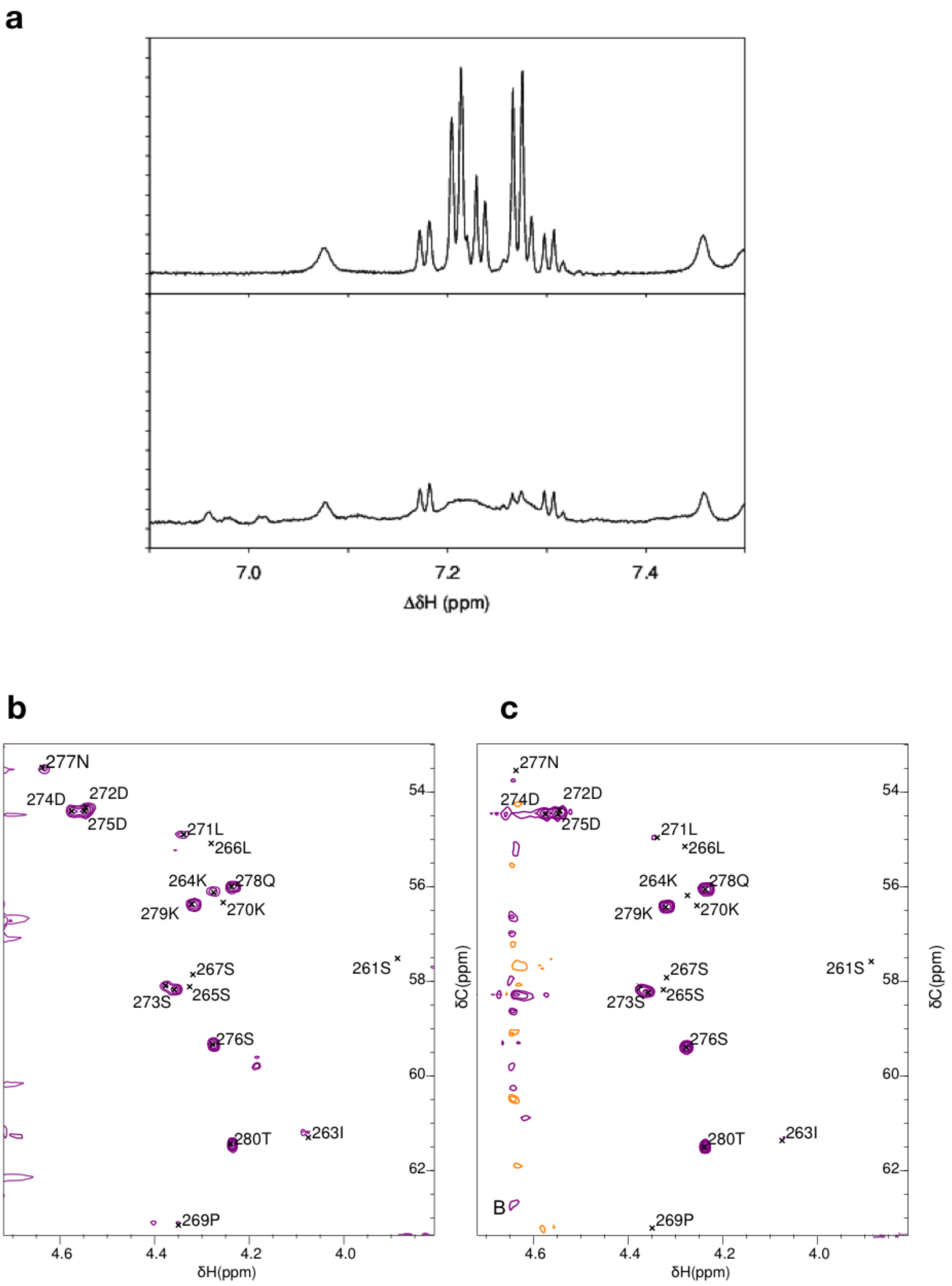

Figure S4. (Related to Figure 5) NMR and PRE studies of UBAP1 peptide in the presence of HD-PTP.

(a) Aromatic regions of ^{1}H NMR spectra of UBAP1$_{C}$, in the absence (upper panel) and presence (lower panel) of HD-PTP$_{CC}$. Narrow line-width signals arising from the sidechain of Phe268 (Hδ# (7.21 ppm), Hε# (7.28 ppm) and Hζ (7.24 ppm)) are clearly visible in the top panel, but absent from the lower one. Present at approximately 25% intensity are signals (7.18, 7.29 and 7.30 ppm) arising from UBAP1 F268 in the *cis* proline isomer of the peptide, which are unaffected by the presence of HD-PTP$_{CC}$. **(b-c)** Effect of electron spin-label at C$_{425}$: Hα region of ^{1}H-^{13}C HSQC of UBAP1$_{C}$ in the presence of spin-labelled variant of HD-PTP$_{CC}$ (10:1 mixture UBAP1$_{C}$:HD-PTP$_{CC}$); **(b)** shows spectrum when spin-label is diamagnetic (reduced with excess ascorbate), and **(c)** shows spectrum when spin-label is paramagnetic.

Supplementary Figure S5

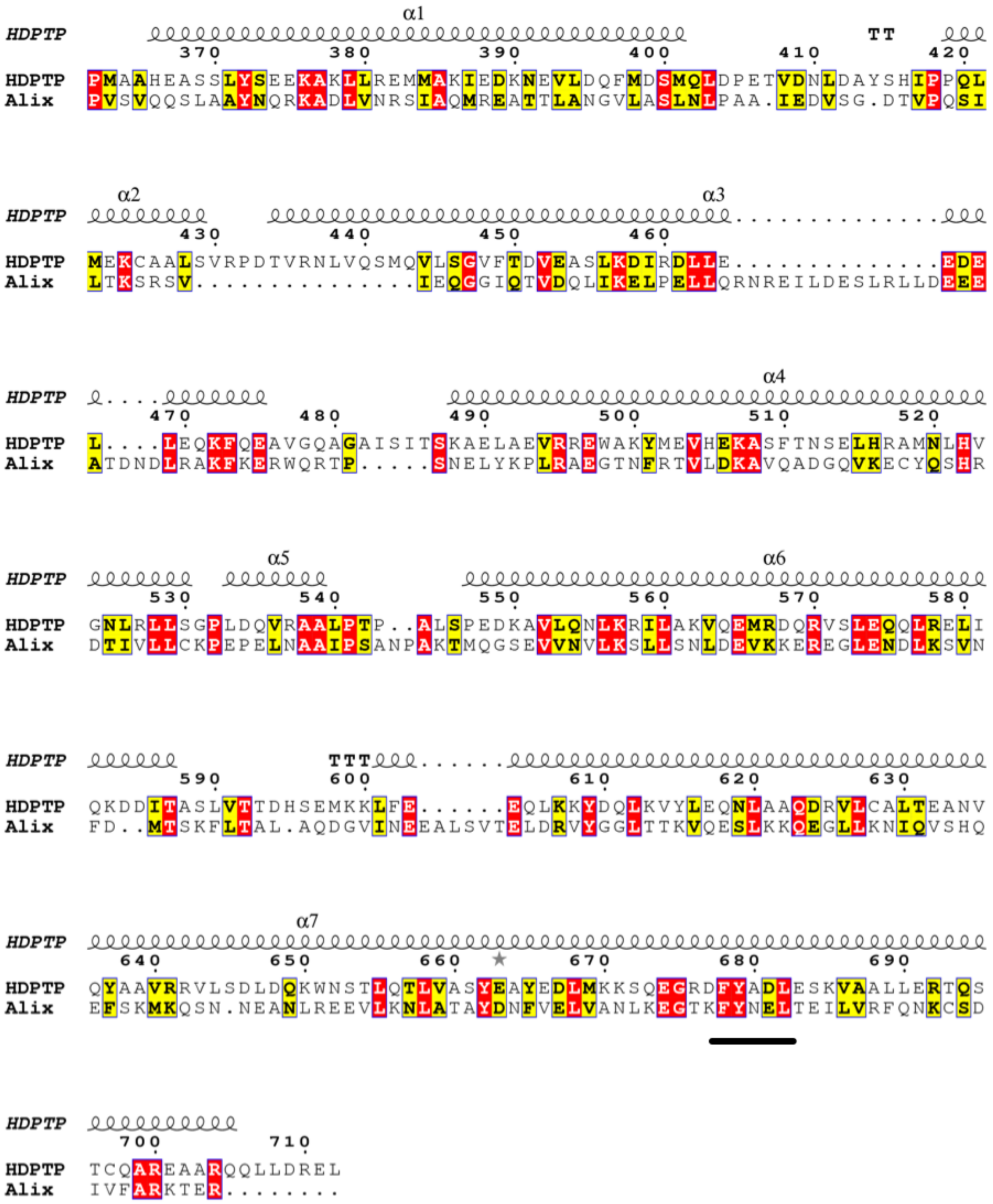

Figure S5. (Related to Figure 6) Sequence alignment of the coiled-coil domains of human HD-PTP and Alix.

Alignment of the sequences of the coiled-coil domain for HD-PTP and Alix. Conservation is highlighted in yellow and identity in red. The conserved FYX_nL motif is underlined in black. Alignment was done using ESPript 3 online server (Robert and Gouet, 2014).

Movie 1. (Related to Figure 4)

A cartoon representation of the coiled coil domain of HD-PTP (HD-PTP$_{CC}$) phosphatase in complex with UBAP1. HD-PTP$_{CC}$ is shown in surface representation and coloured by electrostatic potential. The UBAP1 peptide is shown in stick representation and coloured by atom type. Two electron density maps are displayed for the peptide, an omit map contoured at 2.5σ (green) and a 1σ feature enhanced map (blue) (Afonine et al., 2015). This movie was generated in CCP4MG (McNicholas et al., 2011)

References

Afonine, P. V., Moriarty, N. W., Mustyakimov, M., Sobolev, O. V., Terwilliger, T. C., Turk, D., Urzhumtsev, A. & Adams, P. D. 2015. Fem: Feature-Enhanced Map. *Acta Crystallographica Section D-Structural Biology,* 71**,** 646-666.

Mcnicholas, S., Potterton, E., Wilson, K. S. & Noble, M. E. 2011. Presenting Your Structures: The Ccp4mg Molecular-Graphics Software. *Acta Crystallogr D Biol Crystallogr,* 67**,** 386-94.

Robert, X. & Gouet, P. 2014. Deciphering Key Features In Protein Structures With The New Endscript Server. *Nucleic Acids Res,* 42**,** W320-4.
