# Supplementary figures and images for: Structural Basis for Selective Interaction between the ESCRT Regulator HD-PTP and UBAP1
Source: Structure. 2016 Dec 6;24(12):2115–26. doi: 10.1016/j.str.2016.10.006 (PMC5145805; doi:10.1016/j.str.2016.10.006)

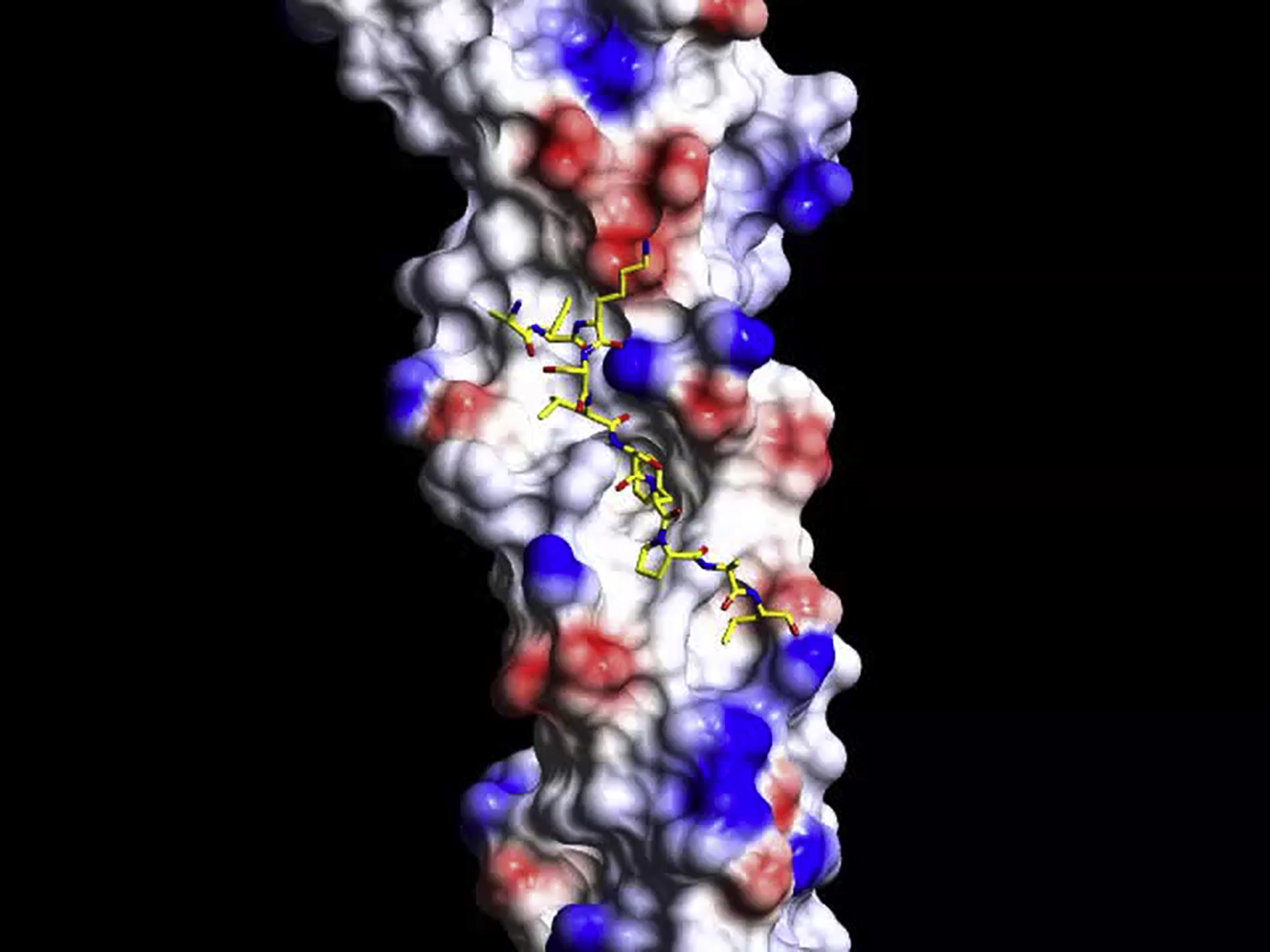

Supplement: Movie S1. UBAP1 Binding Site in HD-PTP [file mmc2.jpg]
