# Supplementary material for: Structural Basis for Selective Interaction between the ESCRT Regulator HD-PTP and UBAP1
Source: Structure. 2016 Dec 6;24(12):2115–26. doi: 10.1016/j.str.2016.10.006 (PMC5145805; doi:10.1016/j.str.2016.10.006)
Supplement: Document S2. Article plus Supplemental Information [file mmc3.pdf]

Structure

Article

Structural Basis for Selective Interaction between the ESCRT Regulator HD-PTP and UBAP1

Graphical Abstract

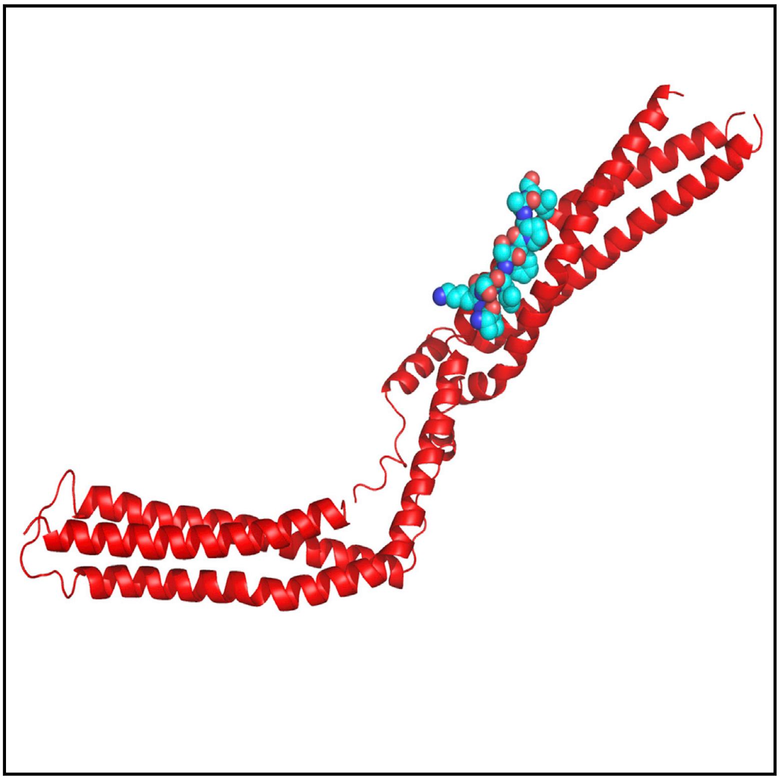

Authors

Deepankar Gahloth, Colin Levy, Graham Heaven, ..., Alistair J. Fielding, Philip Woodman, Lydia Tabernero

Correspondence

philip.woodman@manchester.ac.uk (P.W.), lydia.tabernero@manchester.ac.uk (L.T.)

In Brief

Gahloth, Levy et al. present crystal structures of the coiled-coil domain of the HD-PTP phosphatase, a regulator of ESCRT-dependent sorting, and its complex with the ESCRT-I subunit UBAP1. The structure reveals an open architecture critical for specific interactions and provides insights into how HD-PTP regulates mitogenic receptor sorting.

Highlights

- Crystal structure of the coiled-coil domain of HD-PTP phosphatase with UBAP1
- The open and rigid architecture of HD-PTP is essential for functional recognition
- Conformation and structural determinants define selectivity in Bro1 proteins

Accession Numbers

5LM1
5LM2

Gahloth et al., 2016, Structure *24*, 2115–2126
December 6, 2016 © 2016 The Authors. Published by Elsevier Ltd.
http://dx.doi.org/10.1016/j.str.2016.10.006

CellPress

Structural Basis for Selective Interaction between the ESCRT Regulator HD-PTP and UBAP1

Deepankar Gahloth,[1,3] Colin Levy,[1,3] Graham Heaven,[2] Flavia Stefani,[1] Lydia Wunderley,[1] Paul Mould,[1] Matthew J. Cliff,[1] Jordi Bella,[1] Alistair J. Fielding,[2] Philip Woodman,[1,*] and Lydia Tabernero[1,4,*]
[1]School of Biological Sciences, Faculty of Biology, Medicine and Health
[2]School of Chemistry and Photon Science Institute
University of Manchester, Manchester M13 9PT, UK
[3]Co-first author
[4]Lead Contact
*Correspondence: philip.woodman@manchester.ac.uk (P.W.), lydia.tabernero@manchester.ac.uk (L.T.)
http://dx.doi.org/10.1016/j.str.2016.10.006

SUMMARY

Endosomal sorting complexes required for transport (ESCRTs) are essential for ubiquitin-dependent degradation of mitogenic receptors, a process often compromised in cancer pathologies. Sorting of ubiquinated receptors via ESCRTs is controlled by the tumor suppressor phosphatase HD-PTP. The specific interaction between HD-PTP and the ESCRT-I subunit UBAP1 is critical for degradation of growth factor receptors and integrins. Here, we present the structural characterization by X-ray crystallography and double electron-electron resonance spectroscopy of the coiled-coil domain of HD-PTP and its complex with UBAP1. The coiled-coil domain adopts an unexpected open and rigid conformation that contrasts with the closed and flexible coiled-coil domain of the related ESCRT regulator Alix. The HD-PTP:UBAP1 structure identifies the molecular determinants of the interaction and provides a molecular basis for the specific functional cooperation between HD-PTP and UBAP1. Our findings provide insights into the molecular mechanisms of regulation of ESCRT pathways that could be relevant to anticancer therapies.

INTRODUCTION

Ubiquitination, endocytosis, and degradation of cell-surface receptors constitute a major mechanism of regulation of signal transduction by downregulating receptor availability for interaction with extracellular ligands. Receptor ubiquitination or degradation are often compromised in cancer pathologies, resulting in hyperactivation of signaling pathways promoting cell transformation and tumorigenesis. HD-PTP (His domain protein tyrosine phosphatase; PTPN23) is a non-receptor tumor suppressor phosphatase (Kok et al., 1997; Toyooka et al., 2000) that regulates several ubiquitin-dependent endosomal trafficking processes such as downregulation of EGFR and PDGFRβ signaling (Doyotte et al., 2008; Ma et al., 2015), recycling of Src (Chen et al., 2012; Lin et al., 2011), and degradation of α5β1 integrin (Kharitidi et al., 2015). Consequently, loss of HD-PTP promotes cell proliferation, cell migration, and invasion (Chen et al., 2012; Kharitidi et al., 2015; Lin et al., 2011; Ma et al., 2015; Mariotti et al., 2009). It has been recently reported that HD-PTP haploinsufficiency predisposes mice to tumorigenesis, while hemizygous HD-PTP deletions are observed in many human cancers (Manteghi et al., 2016).

HD-PTP drives the degradation of mitogenic receptors by coordinating their sorting into the multivesicular body (MVB) via specific recruitment of different endosomal sorting complexes required for transport (ESCRTs) (Ali et al., 2013; Stefani et al., 2011). ESCRTs (named ESCRT-0, -I, -II, and -III) are multimeric protein complexes that drive membrane remodeling and scission in a number of important cellular events, including cytokinesis, autophagy, membrane repair, and virus budding (Hurley, 2015). Pathway selectivity is defined by different subsets of ESCRTs and specialized adaptor Bro1 proteins: Bro1 in yeast, HD-PTP and Alix in animals (Bissig and Gruenberg, 2014; Ichioka et al., 2007; Kim et al., 2005). However, the mechanisms by which Bro1 proteins regulate the different ESCRT pathways remain poorly understood. For example, Alix is a general ESCRT-III regulator that supports multiple ESCRT-dependent processes (Bissig and Gruenberg, 2014; Carlton and Martin-Serrano, 2007; Morita et al., 2007; Schmidt et al., 2004; Sun et al., 2015; von Schwedler et al., 2003). In contrast, HD-PTP function is largely restricted to the early endosome (Doyotte et al., 2008; Parkinson et al., 2015) where it acts in close cooperation with ESCRT-0 (Ali et al., 2013) and UBAP1 (ESCRT-I) to downregulate multiple ubiquitinated cargoes (Agromayor et al., 2012; Kharitidi et al., 2015). UBAP1 is an ubiquitin-binding ESCRT-I subunit that acts exclusively in MVB sorting (Stefani et al., 2011; Wunderley et al., 2014) and, like for HD-PTP (Cheng et al., 1998; Toyooka et al., 2000), haploinsufficiency of UBAP1 is linked to nasopharyngeal carcinoma (Qian et al., 2001). UBAP1 is also a risk factor for familial frontotemporal lobar degeneration (Rollinson et al., 2009).

HD-PTP has a multidomain organization that allows coordinated binding to several ESCRTs (Figure 1). Knowledge of the three-dimensional architecture of HD-PTP and its mode of binding to the different ESCRTs is critical for understanding how these functional interactions are regulated. To date, structural

This is an open access article under the CC BY license (http://creativecommons.org/licenses/by/4.0/).

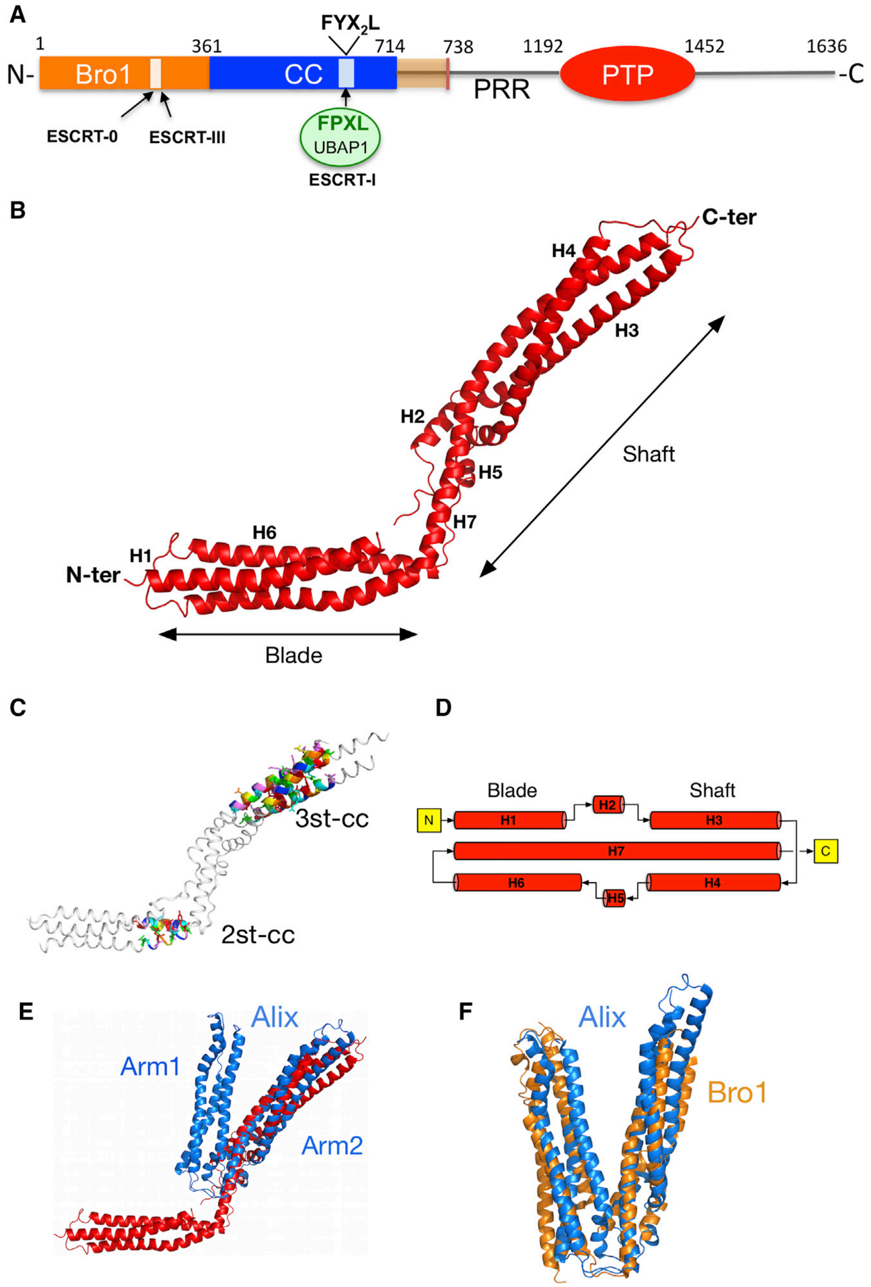

Figure 1. Crystallographic Structure of HD-PTP$_{CC}$

(A) Diagram of HD-PTP domain structure indicating domain boundaries and sites of interaction for ESCRT partners. The position of the conserved FYX$_2$L motif in the CC domain is shown, as well as the FPXL motif in UBAP1.

(B) Cartoon diagram of the HD-PTP$_{CC}$ crystal structure. HD-PTP$_{CC}$ resembles an ice hockey stick, where the N-terminal region represents the blade and the C-terminal region is the shaft. The seven α helices are labeled H1 to H7, with H7 being the central and longest helix extending the whole length of the structure.

(C) Coiled-coil motifs in the HD-PTP$_{CC}$ structure after analysis with SOCKET (Walshaw and Woolfson, 2001). HD-PTP$_{CC}$ contains two canonical coiled coils: one two-stranded coiled coil (2st-cc) in the blade and one three-stranded coiled coil (3st-cc) in the shaft.

(D) Topology diagram of the structure of HD-PTP$_{CC}$ showing the arrangement of the seven α helices.

(E) Superimposition of the HD-PTP$_{CC}$ (red) and Alix$_V$ (blue) structures. The Alix$_V$ crystal structure (PDB: 2OJQ) shows a V-shaped helical protein in a closed conformation, in contrast to the open and extended conformation of HD-PTP$_{CC}$. The two arms in Alix$_V$ are labeled.

(F) Superposition of the structures of Alix$_V$ (blue) and yeast Bro1$_V$ (orange, PDB: 4JIO). Both structures contain two arms joined by flexible loops.

information on HD-PTP is only available for its N-terminal Bro1 domain (Lee et al., 2016; Sette et al., 2011), which binds ESCRT-0 and ESCRT-III (Ali et al., 2013; Doyotte et al., 2008; Toyooka et al., 2000). Structures for the coiled-coil (CC) domains of other Bro1 proteins, Alix and yeast Bro1, have been reported (Fisher et al., 2007; Lee et al., 2007; Pashkova et al., 2013) and they show a V-shaped conformation, with two arms connected by a flexible hinge region (Figure 1). However, the CC domain of HD-PTP has very low homology (17%–19%) to those of Alix or Bro1, suggesting that significant structural disparity may exist. The CC domain of HD-PTP is solely responsible for the interaction with UBAP1, and residue F678 in this domain is essential for binding (Stefani et al., 2011). This Phe residue is part of a FYX$_2$L motif (Figure 1) conserved in all Bro1 proteins (Kimura et al., 2015). In Alix, the FYX$_2$L motif is located on the second arm of the CC domain and mediates binding to viral Gag proteins (Fisher et al., 2007; Lee et al., 2007; Zhai et al., 2008). Paradoxically, the presence of the FYX$_2$L motif in Alix is not sufficient for binding to UBAP1 (Stefani et al., 2011), indicating that other structural determinants may be important to define selectivity.

To address this paradox and to provide new insight into the regulation of ESCRT function by HD-PTP, we have determined the crystal structures of the CC domain of HD-PTP (HD-PTP$_{CC}$) alone and in complex with UBAP1. The structures show an unexpected open conformation of the CC domain, strikingly different from the V domains of Alix and yeast Bro1 (Fisher et al., 2007; Lee et al., 2007; Pashkova et al., 2013). We propose that this open conformation is critical to explain the selective binding of UBAP1 to HD-PTP$_{CC}$ but not to Alix. The HD-PTP$_{CC}$ binding interface with UBAP1 was also analyzed by nuclear magnetic resonance (NMR), paramagnetic relaxation enhancement (PRE), and surface plasmon resonance (SPR), and validated through site-directed mutagenesis and functional cell-based assays, thus establishing the structural basis for the functional cooperation between HD-PTP and UBAP1.

Table 1. Crystallographic Data Collection and Refinement Statistics

	apo-HD-PTP$_{CC}$	HD-PTP$_{CC}$–UBAP1$_{C}$
Data Collection		
Space group	$P2_1$	$P2_1\,2_1\,2_1$
Cell dimensions		
a, *b*, *c* (Å)	53.5, 47.7, 172.7	48.9, 93.3, 102.2
α, β, γ (°)	90.0, 96.0, 90.0	90.0, 90.0, 90.0
Molecules per asymmetric unit	2	1
Resolution (Å)	2.54 (2.63–2.54)[a]	2.55 (2.64–2.55)
R_{merge}	0.1 (0.8)	0.1 (0.8)
$I/\sigma I$	19.3 (3.8)	13.6 (2.5)
Completeness (%)	98 (100)	100 (100)
Redundancy	13.5 (13.8)	6.3 (6.6)
Refinement		
Resolution (Å)	2.54	2.55
No. of reflections	28,609	15,835
R_{work}/R_{free}	21.7/27.8	20.9/25.4
No. of atoms	5,156	2,725
Protein	4,991	2,639
Peptide	NA	73
Water	80	31
B factors		
Protein	49.1	73.6
Peptide	NA	89.7
Water	45.6	56.5
Root-mean-square deviations		
Bond lengths (Å)	0.009	0.003
Bond angles (°)	1.0	0.5

Each structure was determined from one crystal.
[a]Values in parentheses are for the highest-resolution shell.

RESULTS

HD-PTP$_{CC}$ Adopts an Open and Extended Conformation

The crystallographic structure of HD-PTP$_{CC}$ (*apo*-HD-PTP$_{CC}$), determined at 2.5 Å resolution (Table 1), shows an elongated architecture with seven α helices (H1–H7) whose main feature is a central helix of 105 residues (H7) extending the whole length of the molecule (Figure 1B). The overall shape resembles that of an ice hockey stick, in which H1, H6, and the N-terminal region of H7 form the blade, and H2–H5 and the rest of H7 form the shaft (Figure 1B). The maximal dimension of HD-PTP$_{CC}$ is approximately 155 Å from end to end. Analysis of the structure using SOCKET (Walshaw and Woolfson, 2001) identified two canonical coiled-coil motifs: one antiparallel two-stranded coil involving helices H6 and H7 in the blade and an extensive antiparallel, tightly packed three-stranded coil involving helices H3, H4, and H7 in the shaft (Figure 1C). The topology of HD-PTP$_{CC}$ is such that the polypeptide chain crosses three times over the length of the protein (Figure 1D).

The extended shape of HD-PTP$_{CC}$ differs markedly from the V domains of Alix (Alix$_V$) and yeast Bro1 (Bro1$_V$) (Figures 1E and 1F) (Fisher et al., 2007; Lee et al., 2007; Pashkova et al., 2013). These V domains are built from two arms joined by three unstructured loops, forming a flexible hinge (Figures 1E and 1F). By contrast, in HD-PTP$_{CC}$, helix H7 forms a continuous backbone (Figures 1B and 1D) that confers rigidity to the molecule.

HD-PTP$_{CC}$ Shows Limited Local Conformational Flexibility but No Large Structural Rearrangements

Conformational flexibility between the two arms of Alix$_V$ and Bro1$_V$ has been reported (Pashkova et al., 2013; Pires et al., 2009). We investigated the potential flexibility of HD-PTP$_{CC}$ using double electron-electron resonance (DEER) spectroscopy (Figure 2). This technique allows the measurement of dipolar coupling interactions between methanethiosulfonate spin labels (MTSL), covalently attached to cysteines (Figure S1). This was used here to estimate average distances between the labels (Figure 2). Comparison of these distances with those obtained from labels modeled on the HD-PTP$_{CC}$ crystal structure provided information on the conformational dynamics of HD-PTP$_{CC}$. We conducted DEER experiments on wild-type (WT) HD-PTP$_{CC}$ triply labeled at C_{425}, C_{628}, and C_{697}, the three cysteines present in the CC domain, and on doubly labeled mutants where each cysteine in turn had been changed to serine (Figure 2).

Significantly, the C_{425}S mutant, doubly labeled at C_{628}-C_{697} (Figure 2Ai), gave no resolved dipolar coupling, indicating that the spin labels must be separated by more than 7 nm (the maximal mean distance observable in a 6 μs window) (Figure S1). This is consistent with the extended conformation observed in the HD-PTP$_{CC}$ crystal structure, where the distance between labels on C_{628} and C_{697} is 9.9 nm (Figure 2C). Likewise, the interspin distance between C_{425} and C_{697} was estimated to be 6.3 nm (Figures 2Aii and 2Aiii), which matches very well with the 6.0 nm on the crystal structure (Figure 2C).

However, the interspin distance between C_{628} and C_{425} estimated at 4.2 nm (Figures 2Aii and 2Aiv) differs from the distance of 5.4 nm on the crystal structure (Figure 2C), suggesting some local conformational flexibility around C_{425}. This residue is located in helix H2, which is connected by two flanking loops to helices H1 and H3 (Figure 1). These loops are poorly defined in the electron density maps, indicating backbone flexibility in that region. Molecular dynamics simulations around helix H2 also showed that this helix could easily adopt alternative orientations compatible with the DEER results, without requiring large overall conformational changes (Figures 2D and S2).

To confirm the above analyses, an additional Cys residue was engineered at position 521 in H4 and residues C_{425} and C_{697} were changed to Ser. In this case, the mean interspin distance between C_{628} and C_{521} (4.9 nm, Figure 2Av) was in good agreement with the distance on the crystal structure (5.4 nm, Figure 2E). The DEER analysis is therefore consistent with an overall rigid conformation for HD-PTP$_{CC}$ that can accommodate local flexibility without global rearrangements, ruling out a potential mechanism of regulation by large-scale flexibility of the CC domain.

Identification of the Minimal UBAP1 Binding Region to HD-PTP

Specific interaction with UBAP1 is central to the function of HD-PTP (Stefani et al., 2011), and their cooperation is essential to regulate integrin signaling and cell migration (Kharitidi et al.,

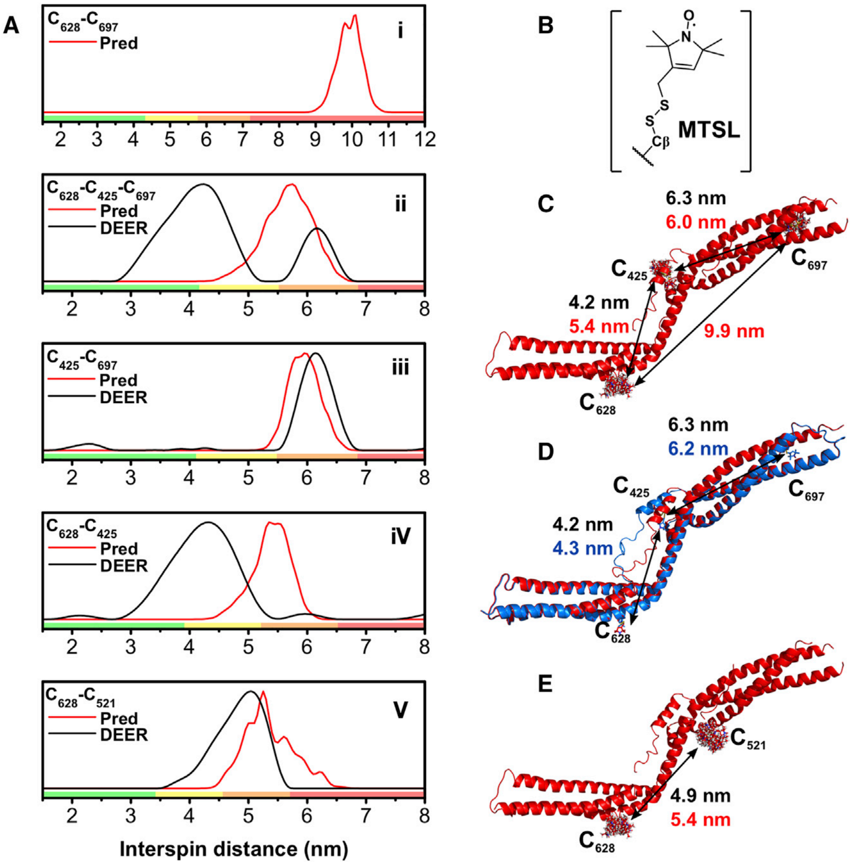

Figure 2. DEER Spectroscopy of HD-PTP$_{CC}$

(A) DEER distance distributions (black) and crystal structure-based predictions (red) are shown for all the experiments using triply labeled HD-PTP$_{CC}$ (ii), or doubly labeled (i, iii, iv, v). Colored boxes from DEERAnalysis (Jeschke et al., 2006) are shown: green is reliable mean, width, and shape; yellow is reliable mean and width; orange is reliable mean; and red indicates long-range distance.

(B) Structure of MTSL-labeled cysteine.

(C) HD-PTP$_{CC}$ structure with modeled MTSL labels at C_{425}, C_{628}, and C_{697}. Mean experimental DEER (black) and predicted (red) distances are shown.

(D) HD-PTP$_{CC}$ structure (red) and model obtained after TAMDS (blue) showing displacement of H2 (where C_{425} is located) and movement of flanking loops. The predicted distance (blue) between labels at C_{425} and C_{628} shows agreement with experimental DEER distances (black).

(E) HD-PTP$_{CC}$ structure with modeled MTSL labels at C_{521} and C_{628}. Mean experimental DEER (black) and predicted (red) distances are shown.

2015). Therefore, we aimed to define this interaction at the molecular level. We previously identified the central region of UBAP1 (122–309) as binding to the CC domain of HD-PTP in a yeast two-hybrid (Y2H) screen (Stefani et al., 2011). We now have confirmed the interaction by co-immunoprecipitation of in vitro translated full-length UBAP1 with bacterially expressed HD-PTP$_{CC}$ (Figure S3). In addition, we have also shown the interaction between UBAP1 and HD-PTP$_{CC}$ in cells. For these experiments, a chimera of FKBP12 fused to HD-PTP$_{Bro1\text{-}CC}$ (FKBP-HD-PTP$_{Bro1\text{-}CC}$) was co-expressed with a chimera of FRB (FKBP12-rapamycin binding) fused to a mitochondrial targeting sequence (mito-FRB). Treatment of cells with rapamycin caused the efficient relocalization of HD- PTP$_{Bro1\text{-}CC}$ to mitochondria. Under these conditions, UBAP1-GFP, but not GFP, also relocated to mitochondria (Figure 3A).

Further truncations of UBAP1 (122–309) identified residues 260–269 as the minimal region for effective binding to HD-PTP (Figure 3B). This region contains an FPXL motif that resembles the YPX$_n$L motif conserved within viral Gag late domains and other substrate proteins that bind to the V domain of Bro1 proteins (Kimura et al., 2015; Lee et al., 2007; Zhai et al., 2008, 2011). The F268S mutation in this motif abolished binding to HD-PTP, while the P269A or L271A mutations had no obvious effect on binding in the Y2H assays (Figures 3A and 3B). This suggests that UBAP1 F268 forms a critical interaction with HD-PTP.

To further characterize this interaction, immobilized HD-PTP$_{CC}$, HD-PTP$_{Bro1\text{-}CC}$, and HD-PTP$_{Bro1}$ were tested in biosensor binding experiments (SPR) using a UBAP1 peptide containing residues 261–280 (UBAP1$_C$) as the analyte. Affinities of UBAP1$_C$ to HD-PTP$_{CC}$ and HD-PTP$_{Bro1\text{-}CC}$ were similar, with dissociation constants K_d of 66.3 μM and 31.9 μM, respectively (Figures 3C and 3D). No binding was observed to HD-PTP$_{Bro1}$ (Figure 3D), thus confirming that the main UBAP1-binding region is within the CC domain, and that the conformation of the CC domain is functionally competent, both on its own and in the presence of the Bro1 domain.

Mapping the Molecular Interface between HD-PTP$_{CC}$ and UBAP1$_C$

In order to define the molecular interactions, we determined the crystal structure of HD-PTP$_{CC}$ in complex with UBAP1$_C$ at 2.5 Å resolution (Table 1). UBAP1$_C$ adopts a rather extended conformation and binds to the shaft, near the core of the three-strand coiled coil, between H4 and H7 (Figure 4A and Figure 1C). Only residues 262–271 from UBAP1$_C$ were clearly identified in the electron density maps (Movie S1) and the side chains of I263, L266, F268, P269, and L271 are in direct contact with HD-PTP$_{CC}$ (Figure 4). Residues 272–280 were disordered and not visible, probably because of weaker affinity. Interactions between UBAP1$_C$ and HD-PTP$_{CC}$ are mainly hydrophobic, with hydrogen bonds only present between K671 on HD-PTP$_{CC}$ and the main chain of I263, K264, and L266 on UBAP1$_C$ (Figure 4B).

We confirmed the molecular interface between HD-PTP$_{CC}$-UBAP1$_C$ using NMR by comparing the ^{1}H ^{13}C-heteronuclear single quantum coherence (HSQC) spectra of UBAP1$_C$ in the absence and presence of HD-PTP$_{CC}$. Significant peak broadening was only observed for residues 261–271 in the presence of HD-PTP$_{CC}$ (Figures 5A, 5B, and S4), consistent with the interactions observed in the crystal structure, and with the minimal UBAP1 binding region identified by Y2H (Figure 3B).

The binding interface in HD-PTP$_{CC}$ is defined by three hydrophobic pockets (A–C in Figure 4) and the conserved FYX$_2$L motif

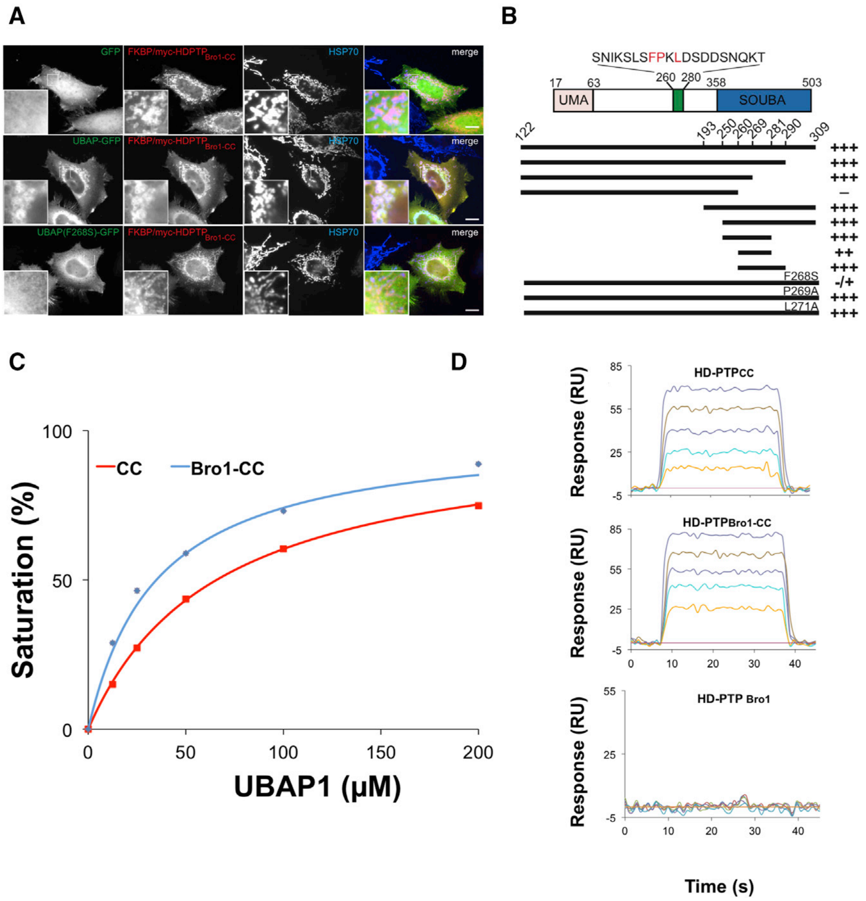

Figure 3. Minimal Binding Region in UBAP1 Responsible for Interaction with HD-PTP

(A) HeLa cells transfected with FKBP-HD-$PTP_{Bro1\text{-}CC}$-myc, Mito-FRB, and either GFP, UBAP1-GFP wild-type, or UBAP1-GFP F268S were treated with rapamycin and imaged by immunofluorescence for GFP, myc, and mitochondrial Hsp70. Scale bar, 10 μm. Upon treatment, UBAP1-GFP translocates to mitochondria colocalizing with FKBP-HD-$PTP_{Bro1\text{-}CC}$ but not GFP or UBAP1-GFP F268S.

(B) Yeast two-hybrid interactions between UBAP1 fragments and HD-$PTP_{Bro1\text{-}CC}$. +/− symbols indicate the degree of growth.

(C) Biosensor binding isotherms for the different HD-PTP constructs to $UBAP1_C$. Affinity to HD-PTP_{CC}, and HD-$PTP_{Bro1\text{-}CC}$ was similar, with dissociation constants K_d of 66.3 ± 0.35 μM and 31.9 ± 0.85 μM, respectively. Error bars represent the SEM, n = 3.

(D) Biosensor sensograms for the immobilized HD-PTP constructs binding to $UBAP1_C$ peptide, showing that the CC domain is responsible for binding since HD-PTP_{Bro1} fails to bind to $UBAP1_C$.

plays a critical role in the interaction. The aryl ring of HD-PTP_{CC} F678 forms a wall that divides pockets B and C and contributes to multiple hydrophobic interactions with $UBAP1_C$ (Figure 4). Pocket A is large and shallow and accommodates $UBAP1_C$ residues I263 and L266. This pocket is lined by T511, L515, A518 (H4 helix) and A664, L668, K671 (H7 central helix). Pocket B is deep and narrow and accommodates $UBAP1_C$ F268 and P269. This pocket includes A508, T511 (H4), L445 (H3), and G675, F678 and Y679 (H7), forming extensive contacts with the aryl ring of F268. Pocket C accommodates L271 and includes V504 (H4) and L682 (H7). This pocket is closed off by a high rim provided by the side chains of K500, Y501 (H4), and K685 (H7) (Figure 4B).

The tight fit of F268 in the B pocket explains the key role of this residue for binding to HD-PTP, as shown by Y2H (Figure 3). Conversely, $UBAP1_C$ P269 and L271 show fewer contacts with HD-PTP_{CC}: V504 and F678 near pocket B, and V504 and L682 in pocket C, respectively (Figure 4). The minor contribution of these two residues to the binding interface explains the weaker phenotype observed for the single mutations P269A and L271A (Figure 3), particularly in the presence of F268, which is the main anchor into the binding site. Hydrogen bonds are only present between the side chain of HD-PTP_{CC} K671 in pocket A and the main chain of I263, K264, and L266 in $UBAP1_C$ (Figure 4B).

Pocket A is partially occluded in the structure of apo-HD-PTP_{CC} (Figure 4E) by the side chains of K671, D667, and E514. Binding of $UBAP1_C$ thus requires rearrangement of these three side chains (Figure 4F). In the apo-HD-PTP_{CC} structure, D667 and K671 form a salt bridge, and the carboxyl group of E514 is about 6.4–7 Å to D667 and K671 (Figure 4F). Upon $UBAP1_C$ binding, E514, D667, and K671 side chains are displaced breaking the K671-D667 salt bridge and opening the A pocket. K671-Nζ then forms hydrogen bonds with the C=O groups of I263, K264, and L266 in $UBAP1_C$ (Figure 4B).

The orientation of $UBAP1_C$ in the HD-PTP_{CC} binding site was validated in PRE experiments with MTSL labels at C_{425} and C_{628}. Dipolar interaction with the electron spin label causes an additional component of the transverse relaxation of the NMR signal, which has strong distance dependence with maximal effect below 40 Å. The paramagnetic enhancement in this case is dominated by the spin label attached to C_{425}, which is closer to the $UBAP1_C$ binding site. Consistent with this, NMR resonances from the N-terminal end of the $UBAP1_C$ showed enhancement of their relaxation properties and therefore loss of peak intensity in the presence of the MTSL label (oxidized form), whereas those from the region beyond L271 (>40 Å from the label) did not (Figure 5C), thus confirming the orientation of $UBAP1_C$ at the binding site.

Structural Basis for the Specific Functional Interaction of UBAP1 with HD-PTP

All Bro1 proteins contain a conserved FYX_2L motif essential for binding to their substrate proteins, which contain a reciprocally conserved YPX_nL motif (Kimura et al., 2015). Several structures of Alix in complex with retroviral Gag late-domain peptides (Fisher et al., 2007; Lee et al., 2007) (Figure 6) have shown that these conserved motifs interact with each other, thus confirming their functional importance. The FY pair in the FYX_2L motif in Alix stacks against Y in the Gag peptide YPX_nL motif, thereby serving

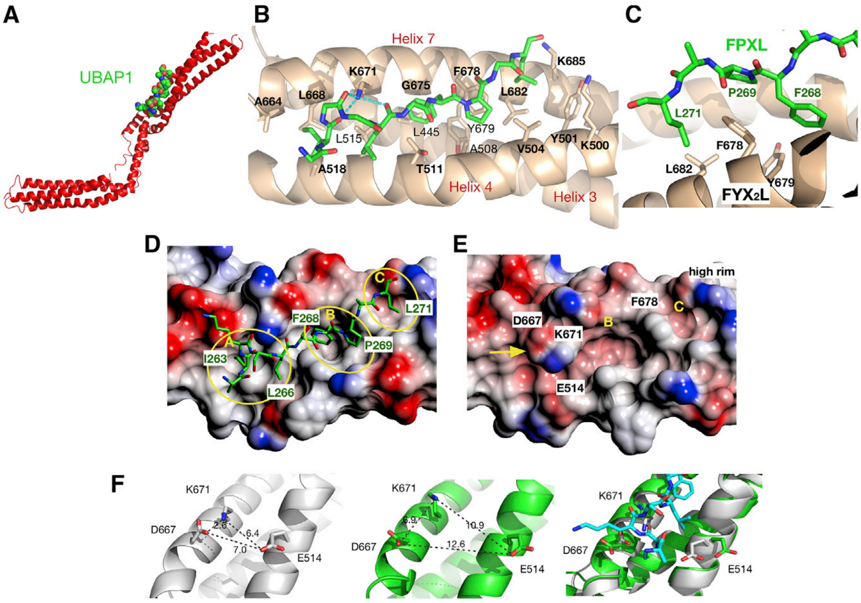

Figure 4. Crystal Structure of the HD-PTP$_{CC}$-UBAP1$_{C}$ Complex and Analysis of the Binding Interface

(A) Structure of HD-PTP$_{CC}$ (red cartoon) in complex with UBAP1$_{C}$ (space-filling) showing that the binding site is located in the middle of the shaft region.

(B) Overview of UBAP1$_{C}$ (sticks) binding site in HD-PTP. The three helices in HD-PTP that form the binding site in the shaft are labeled (red) and represented as cartoons. Residue side chains that participate in interactions with UBAP1$_{C}$ are shown as sticks and labeled (black). Residue K671 forms hydrogen bond interactions (cyan dashed lines) with three carbonyl oxygens in the UBAP1$_{C}$ peptide (sticks).

(C) Detail of the UBAP1$_{C}$ binding site (pocket B) showing the HD-PTP$_{CC}$ residues in the conserved FYX$_{n}$L motif: F678, Y679, and L682 forming hydrophobic interactions with the UBAP1c F268, P269, and L271 in the FPXL motif.

(D) Electrostatic surface of HD-PTP$_{CC}$ at the UBAP1$_{C}$ binding site showing three main pockets A–C (yellow circles) that accommodate the peptide. UBAP1$_{C}$ is shown as sticks. Residues in UBAP1$_{C}$ that interact with HD-PTP$_{CC}$ are labeled: I263, L266 bind to pocket A; F268 and P269 bind to pocket B; L271 binds to pocket C.

(E) Electrostatic surface of the structure of *apo*-HD-PTP$_{CC}$ showing that pocket A is occluded by the side chains of K671 and D667, forming a salt bridge (yellow arrow).

(F) Detail of the structure of *apo*-HD-PTP$_{CC}$ (white ribbon left) and HD-PTP$_{CC}$ in complex with UBAP1$_{C}$ (green ribbon center). In the complex, the side chains of K671 and D667 are shifted (middle panel) and the salt bridge that they form in the *apo* structure (left panel) is lost. The side chain of E514 is also displaced, making room to accommodate the peptide in pocket A (right panel).

as the main anchoring point for their interaction (Fisher et al., 2007; Lee et al., 2007). Similar binding motifs are found in HD-PTP (FYADL) and UBAP1 (FPTL), yet the binding of UBAP1 to HD-PTP is surprisingly highly selective (Stefani et al., 2011). In our HD-PTP$_{CC}$-UBAP1$_{C}$ structure, the FY pair (F678, Y679) in HD-PTP forms stacking interactions with F268 in the FPXL motif of UBAP1$_{C}$ (Figure 4). The critical importance of these residues for binding was confirmed by SPR, where both the HD-PTP F678D and UBAP1 F268S mutations abolished binding (Figure 7A).

Despite similarities in the molecular recognition motifs, important differences are apparent when comparing the structures of HD-PTP$_{CC}$-UBAP1$_{C}$ and Alix$_{Bro1-V}$-Gag peptide complexes. First, the binding site of UBAP1$_{C}$ is displaced with respect to that of the Gag peptides (Figure 6A): UBAP1$_{C}$ occupies pocket A in HD-PTP$_{CC}$, which is poorly conserved in Alix and does not participate in Gag peptide binding (Figures 6 and S5). This displacement is possible because the architecture of HD-PTP$_{CC}$ offers an open, extended interface suitable to accommodate UBAP1$_{C}$. In contrast, in all the complexes of Alix$_{Bro1-V}$ with Gag peptides, both arms of the V domain form an apex that makes pocket A inaccessible, thus preventing binding due to steric hindrance (Figure 6). Second, in Alix, there is a hydrophobic binding groove that accommodates the Gag peptides and extends beyond pocket C. In HD-PTP$_{CC}$, the side chains of K500, Y501, and K685 form a high rim at the edge of this pocket (Figures 4 and 6), and the three-stranded coiled coil results in tight packing between H4 and H7, leaving no room for a groove. These structural features also explain why UBAP1$_{C}$ is displaced toward pocket A with respect to the position of the Gag peptides on Alix (Figure 6).

We believe that the differences in the architecture and binding interface features observed between Alix and HD-PTP are critical in determining ligand-binding selectivity. We confirmed this by showing that in solution, Alix$_{V}$ does not bind to the UBAP1$_{C}$ peptide, and that a Gag peptide (SIV-GAG) does not bind to HD-PTP$_{CC}$ (Figure 7B), mirroring the lack of binding of full-length UABP1 to Alix$_{Bro1-V}$ (Stefani et al., 2011). The conserved FYX$_{2}$L motif is therefore necessary for binding but insufficient to determine specific selectivity between Bro1 proteins and their biological partners. Instead, both the overall architecture and local structural determinants appear to be key in defining molecular recognition and binding specificity.

Functional Validation of HD-PTP-UBAP1 Interactions

The HD-PTP$_{CC}$-UBAP1$_{C}$ interface was validated by RNAi rescue experiments using HD-PTP$_{Bro1-CC}$ and mutations at the binding site. In normal cells, EGFR that has been activated by EGF passes through the endosomal pathway and is degraded within lysosomes. In contrast, cells depleted of HD-PTP are characterized by the accumulation of ligand-activated EGFR in highly clustered early endosomes that label strongly for protein-ubiquitin conjugates (Doyotte et al., 2008). We have previously shown that reintroduction of HD-PTP$_{Bro1-CC}$ is sufficient to rescue these trafficking defects and represents the minimal functional region of HD-PTP (Doyotte et al., 2008). HeLa cells depleted of HD-PTP and pulsed for 3 hr with EGF, showed intense ubiquitin labeling on cytoplasmic clusters (Figure 7C), which co-labeled with the endosomal marker EEA1 as previously reported (Doyotte et al., 2008) (data not shown). As expected, transfection of these cells with WT HD-PTP$_{Bro1-CC}$ restored a WT phenotype, in which

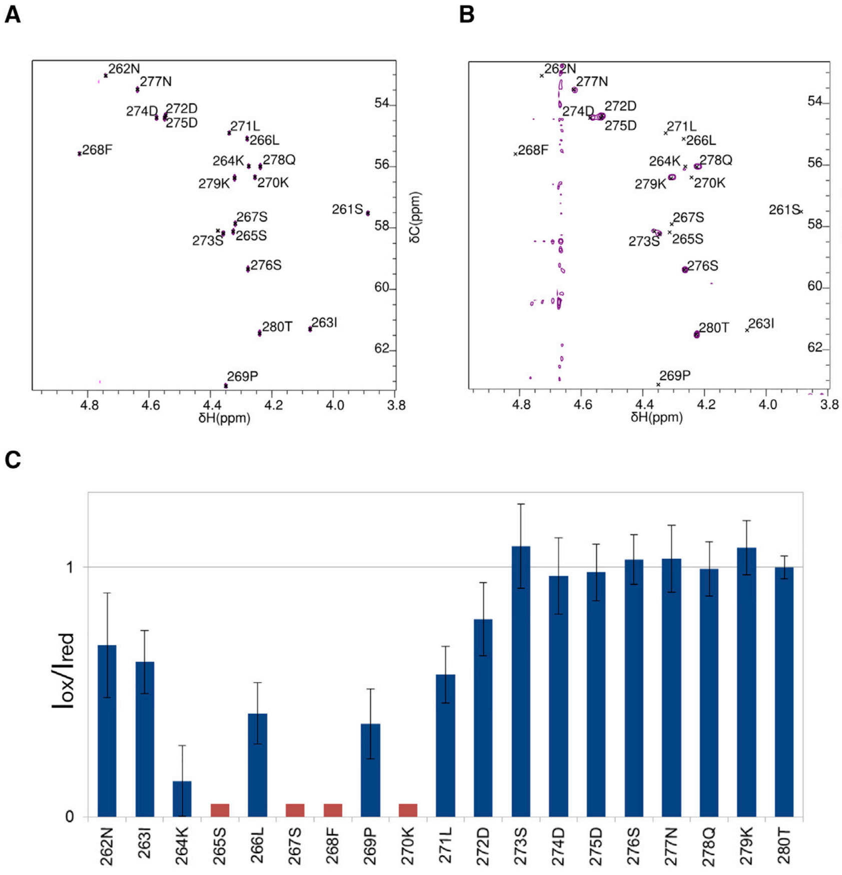

Figure 5. NMR Analysis of the HD-PTP$_{CC}$-UBAP1$_C$ Binding Interface

(A) Hα region of ^{1}H^{13}C-PUSH-HSQC of UBAP1$_C$ (natural abundance ^{13}C) in PBS-D_2O showing the resonance assignment.

(B) Hα region of ^{1}H^{13}C-HSQC of UBAP1$_C$ (natural abundance ^{13}C) in the presence of sub-stoichiometric HD-PTP$_{CC}$. The D_2O content in these samples was approximately 80% and significant intensity arises from H_2O, compromising interpretation of signals between 4.8 and 4.6 ppm. Residues close in sequence to UBAP1 F268 are significantly broadened by interaction with HD-PTP$_{CC}$. Residues C-terminal of UBAP1 D272 are less affected by the presence of HD-PTP$_{CC}$, and therefore are identified as not being involved in the binding site.

(C) Per residue mean peak intensity ratios between UBAP1$_C$ samples containing paramagnetically (I_{ox}) and diamagnetically (I_{red}) labeled HD-PTP$_{CC}$ (C_{425}, C_{628}), indicating the extent of paramagnetic relaxation enhancement. For some resonances, intensity ratios are low because of the line broadening induced by binding, and these are marked by red bars in the chart.

Error bars are SDs estimated from the noise level in the HSQC spectra.

ubiquitinated proteins were evenly distributed throughout the cell (Figure 7C).

In contrast, the HD-PTP$_{Bro1\text{-}CC}$ F678D mutant was unable to rescue depletion of HD-PTP, with both transfected and untransfected cells displaying strong ubiquitin labeling on cytoplasmic inclusions (Figure 7C). The HD-PTP$_{Bro1\text{-}CC}$ K671A mutant failed to completely rescue a WT phenotype, but showed milder defects than F678D (Figure 7D). Although K671 forms hydrogen bond interactions with UBAP1$_C$ (Figures 4 and 6), its overall contribution to binding affinity is clearly less critical when compared with the contribution of the extensive hydrophobic interactions of F678. These data extend our previous findings (Stefani et al., 2011), confirm the functional significance of the interface observed in the crystal structure, and, importantly, demonstrate that binding of HD-PTP to UBAP1 is essential for correct sorting of activated EGFR, since Alix does not function in this ESCRT pathway.

DISCUSSION

The close functional cooperation between HD-PTP and UBAP1 is physiologically crucial. Disruption of this cooperation by ablation or genetic defects leads to cancer, altered cell migration, and neurological pathologies (Cheng et al., 1998; Kharitidi et al., 2015; Qian et al., 2001; Rollinson et al., 2009). Our findings explain the structural basis for the interaction between HD-PTP and UBAP1 and reveal why UBAP1 binding is selective to HD-PTP despite conservation of the FYX$_2$L binding motif across other Bro1 proteins. In addition, the remarkably different architecture of the HD-PTP CC domain compared with other Bro1 proteins provides further insights into the assembly of specialized ESCRTs at the endosome that drive downregulation of cell-surface receptors.

Our data show that HD-PTP$_{CC}$ adopts an open and extended architecture, where the CC domain maintains a rigid conformation by virtue of the long central α helix. This structure contrasts with the V-shaped CC domains of Alix and yeast Bro1 (Fisher et al., 2007; Lee et al., 2007; Pashkova et al., 2013), and is consistent with the low homology found between HD-PTP$_{CC}$ and these V domains.

We revealed the structural determinants for specific interaction between HD-PTP and UBAP1 by X-ray crystallography, NMR, and mutagenesis. Our results confirm that the conserved HD-PTP residue F678 in the FYX$_2$L motif is essential for binding to UBAP1. Furthermore, key hydrophobic interactions between UBAP1 residues F268, P269, and L271 (in the FPXL motif) and HD-PTP residues F678, Y679, and L682 (in the FYX$_2$L motif) form the core of the binding interface. We demonstrated the critical importance of F268 and F678 residues by mutagenesis, binding analysis, and functional cellular assays.

Our findings match the reported roles of Alix F676, yeast Bro1 F687, and Rim20 F623 in binding to Gag late domains, Rfu1, and Rim101, respectively (Fisher et al., 2007; Kimura et al., 2015; Lee et al., 2007). Paradoxically, conservation of the FYX$_2$L motif in Bro1 proteins and that of the YPX$_n$L motif in their biological targets does not result in promiscuous interactions (Kimura et al., 2015; Stefani et al., 2011). Our studies bring new insight into understanding this paradox. Significant differences in the sequence, local structural features, and hydrophobicity of the

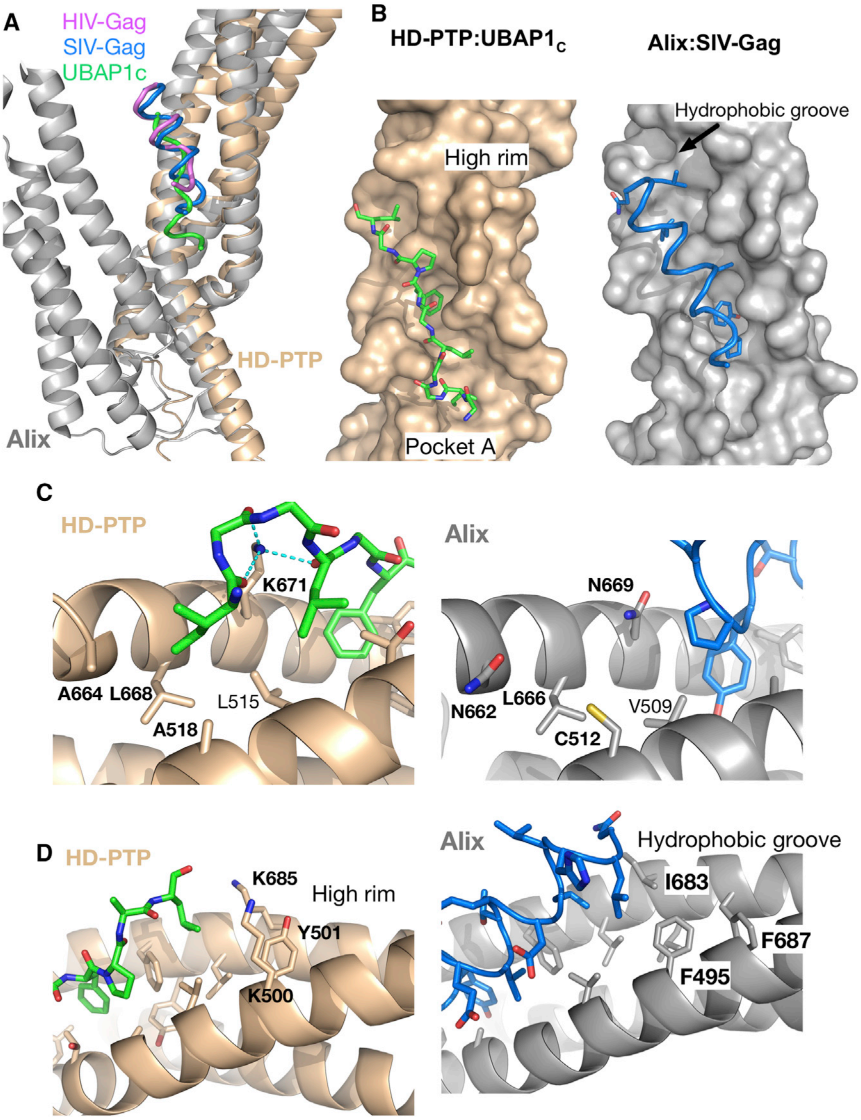

Figure 6. Comparison of the Binding Sites in the HD-PTP$_{CC}$-UBAP1$_C$, and Alix-Gag Peptide Complexes

(A) Structures of Alix (gray ribbon) in complex with HIV-Gag (magenta) and SIV-Gag (blue), superimposed on the structure of HD-PTP$_{CC}$ (gold ribbon) in complex with UBAP1$_C$ (green). The UBAP1$_C$ binding site is displaced with respect to the Gag peptides that bind along a distal hydrophobic groove not conserved in HD-PTP. Conversely, the closed conformation of the Alix$_V$ domain prevents full access to the region near the apex, making pocket A inaccessible.

(B) Enlarged view into the binding site of UBAP1c (sticks) in HD-PTP$_{CC}$ (gold surface) and of the complex of SIV-Gag peptide (blue ribbon) in Alix$_V$ (gray surface). Only residues that interact are shown. Pocket A and the high rim in HD-PTP that closes off pocket C are labeled. In the complex of Alix$_{Bro1-V}$ with SIV-Gag, the binding site extends toward a hydrophobic groove beyond pocket C, but no binding occurs in the equivalent to pocket A in HD-PTP.

(C) Detailed view of pocket A in HD-PTP (left, gold ribbon) and Alix (right, gray ribbon) showing the side chains in sticks. The lack of sequence conservation in this pocket together with steric hindrance may explain the lack of interactions of the SIV-Gag peptide with Alix (right), whereas in HD-PTP, pocket A provides several hydrophobic interactions with residues in the UBAP1$_C$ peptide (labeled) in addition to hydrogen bonds with K671 in HD-PTP.

(D) Detail view of pocket C showing the HD-PTP residues that form the high rim that closes off pocket C (left) and prevents binding of UBAP1$_C$ peptide beyond this point. In contrast, SIV-Gag forms interactions along a hydrophobic groove in Alix (right) that extends beyond pocket C.

UBAP1$_C$ binding site in HD-PTP, compared with that of the Gag peptide site in Alix, are critical to determine specificity. Furthermore, the open architecture of HD-PTP$_{CC}$ is essential to enable binding of UBAP1$_C$ to pocket A, which is inaccessible in the V-shaped structure of Alix observed in the complexes with Gag peptides. Thus, the conformational differences may provide additional molecular determinants for the binding selectivity that we observe.

Alix$_V$ has been reported to be flexible in solution and therefore able to adopt a more open conformation (Pashkova et al., 2013; Pires et al., 2009). However, we found no evidence that Alix binds the UBAP1$_C$ peptide in our binding studies in solution, or of binding to the full-length UBAP1 by Y2H (Stefani et al., 2011).

We hypothesize that differences in the overall architecture of Bro1 proteins, and in the local features at the binding site, combine to elicit exquisite functional selectivity for ESCRT pathway regulation. This is certainly the case for HD-PTP and Alix. Whether these principles apply to other Bro1 proteins and their targets will require further high-resolution analyses of their complexes.

We found that there is no evidence for large-scale flexibility of HD-PTP$_{CC}$, as has been suggested for the V domains of Alix (Fisher et al., 2007; Pires et al., 2009) and yeast Bro1 (Pashkova et al., 2013). In addition, the open conformation exhibited by HD-PTP$_{CC}$ most likely extends to the entire Bro1-CC region, since the UBAP1$_C$ peptide binds equally well to HD-PTP$_{CC}$ and HD-PTP$_{Bro1-CC}$. Indeed, HD-PTP$_{Bro1-CC}$ is functionally competent as demonstrated by its binding to UBAP1 in cells and its ability to rescue defects in EGFR sorting caused by RNAi-mediated depletion of endogenous HD-PTP. Altogether, these new findings point to fundamental differences in how HD-PTP and Alix are regulated and how they control ESCRT function to define pathway diversity at different sub-cellular locations.

EXPERIMENTAL PROCEDURES

Cloning, Protein Expression, and Purification

Constructs for HD-PTP$_{Bro1}$ (1–361), HD-PTP$_{CC}$ (363–712), and Alix$_V$ (358–702) were subcloned into a pNIC28a-Bsa4 vector (gift from Opher Gileadi; Addgene no. 26103). HD-PTP$_{Bro1-CC}$ (1–714) was cloned into a pET28a vector with restriction sites Nde1 and Xho1. Point mutants were generated by quick-change

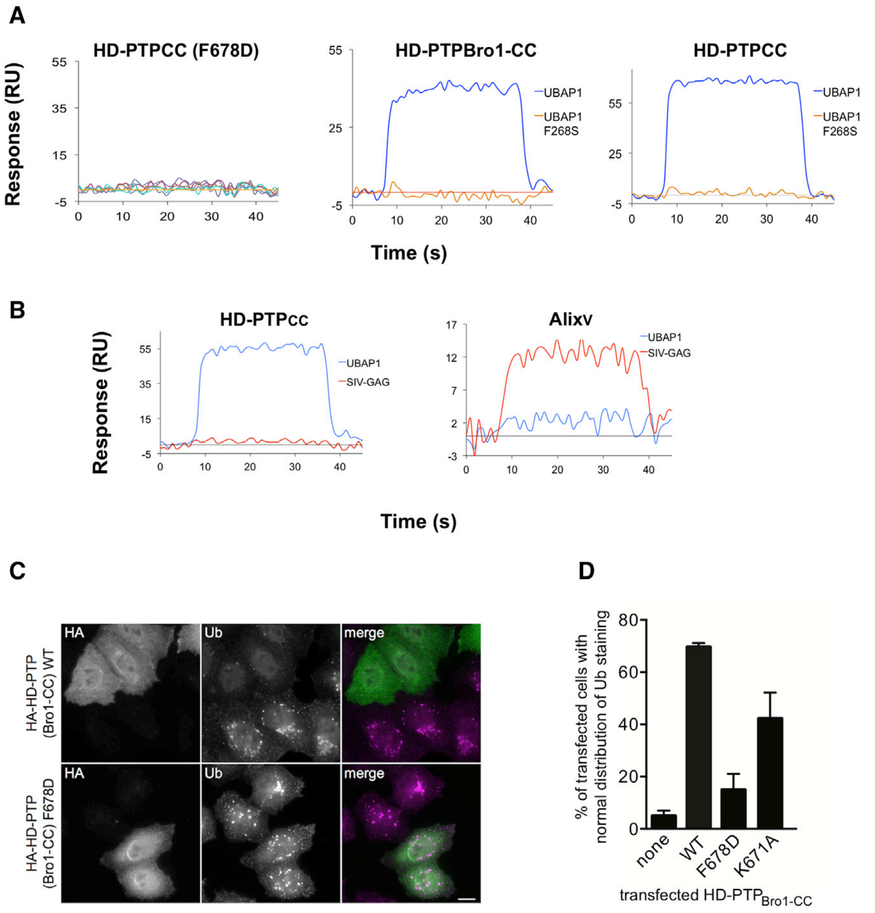

Figure 7. Biochemical and Functional Validation of the UBAP1$_C$ Binding Interface with HD-PTP

(A) Biosensor sensograms of the binding of HD-PTP$_{CC}$ containing the F678D mutation to UBAP1$_C$ peptide (left) and of binding of HD-PTP$_{Bro1\text{-}CC}$ and HD-PTP$_{CC}$ to UBAP1$_C$ peptide containing the F268S substitution (center and right). Both mutations abolished interaction.

(B) Binding to the CC domain is selective despite conservation of the FYX$_2$L motif in both Alix and HD-PTP$_{CC}$: UBAP1$_C$ does not bind to Alix and the SIV-Gag peptide does not bind to HD-PTP$_{CC}$.

(C) Cells depleted of HD-PTP were transiently transfected with HA-tagged HD-PTP$_{Bro1\text{-}CC}$ and stimulated with EGF for 3 hr before fixing and staining with anti-ubiquitin (Ub). Cells transfected with WT HD-PTP$_{Bro1\text{-}CC}$ display even ubiquitin distribution throughout the cell, while untransfected cells or those transfected with the F678D mutant display very strong accumulations on cytoplasmic inclusions. These co-label with endosomal markers as previously reported (Doyotte et al., 2008) (data not shown). Scale bar, 10 μm.

(D) Scoring of rescue experiments. Cells transfected as indicated were scored for normal ubiquitin distribution. One hundred cells from three independent experiments were counted, and SDs between these experiments are shown. Two-way ANOVA analysis: F678D versus WT, p = 0.0025; K671A versus WT, p = 0.043; K671A versus F678D, p = 0.002.

primers using Phusion DNA polymerase (New England Biolabs). Constructs were expressed in BL21(DE3) *Escherichia coli* using 0.1 mM isopropyl β-D-1 thiogalactopyranoside induction overnight at 20°C. Cells were lysed in 20 mM HEPES (pH 7.0), 500 mM NaCl, 10 mM imidazole, 2 mM phenylmethylsulfonyl fluoride by sonication, and the supernatant was clarified by centrifugation at 12,400 × *g* for 1 hr. Proteins were purified by affinity chromatography using nickel-beads (QIAGEN) pre-equilibrated in binding buffer (20 mM HEPES, 500 mM NaCl, 10 mM imidazole [pH 7.4]) followed by anion-exchange chromatography with a Mono Q 5/50 GL column (GE Healthcare) in 20 mM HEPES (pH 7.4), 2 mM EDTA, 2 mM DTT, and super elongation complex (SEC) using a Superdex200 column (GE Healthcare) in the same buffer. Incorporation of L-selenomethionine was achieved by growing a culture in M9 minimal medium (Molecular Dimensions) supplemented with essential amino acids (100 mg/L each) and selenomethionine (80 mg/L).

Crystallization and Structure Determination

For crystallization, the His$_6$ tag of HD-PTP$_{CC}$ was removed by cleavage with tobacco etch virus protease followed by nickel-affinity chromatography and further purification as above. *apo*-HD-PTP$_{CC}$ (11 mg/mL) was crystallized in 0.1 M Bis-Tris (pH 6.0), 0.1–0.2 M Na-formate, and 13%–15% PEG$_{3350}$ at 21°C. Crystals of the HD-PTP$_{CC}$-UBAP1$_C$ complex were obtained by mixing HD-PTP$_{CC}$ (1 mg/mL) with 1 mM UBAP1 peptide and concentrated to 11 mg/mL from a reservoir solution of 0.2 M KSCN, 20% PEG$_{3350.}$ All crystals were cryo-protected in perfluoropolyether cryo oil (Hampton Research) prior to freezing in liquid nitrogen. Data were collected at I02 and I03 beamlines in Diamond Light Source (UK) and processed with XDS (Kabsch, 2010). The structure of *apo*-HD-PTP$_{CC}$ was determined by selenium single-wavelength anomalous diffraction (Se-SAD) using PHENIX AutoSol, and the initial model built using PHENIX AutoBuild (Adams et al., 2010) and COOT (Emsley et al., 2010) and refined using PHENIX Refine. The structure of HD-PTP$_{CC}$-UBAP1$_C$ was determined by molecular replacement using the *apo* structure as the search model using PHENIX Phaser and model building and refinement in COOT and PHENIX Refine.

EPR-DEER Measurements

Proteins were purified as describe above. A 15-fold molar excess of MTSL (Toronto Research Chemicals) was mixed with the protein sample and incubated at 4°C overnight followed by SEC on a Superdex200 column (GE Healthcare) equilibrated in 20 mM HEPES, 250 mM NaCl, 5 mM EDTA (pH 7.4). Labeling was confirmed by mass spectrometry. All mutants and the WT were checked by circular dichroism to confirm the secondary structure (Figure S1). DEER samples were prepared by buffer exchange into deuterated buffer (20 mM HEPES, 250 mM NaCl, in D_2O [pD 7.4]) with 30% (v/v) glycerol-d$_8$ to a final protein concentration of 60 μM and flash frozen in 4 mm quartz tubes (Wilmad). The four-pulse DEER experiments were carried out on a pulsed ELEXSYS E580 (9 GHz) spectrometer (Bruker), cooled to 50 K with a continuous-flow helium CF935 cryostat and an isothermal calorimetry 502 temperature control system (Oxford Instruments) and analyzed with DEERAnalysis 2013.2 (Jeschke et al., 2006). Distance distribution predictions were calculated using MMM 2013.2 (Multiscale Modeling of Macromolecular systems) (Polyhach et al., 2011).

Torsion-Angle Molecular Dynamics

CNS (Crystallography & NMR System, version 1.3) (Brunger et al., 1998) was used to run torsion-angle molecular dynamics simulations (TAMDS) of HD-PTP$_{CC}$ models with MTSL spin-labeled side chains at selected positions. Torsional angle flexibility was limited to the MTSL side chains and the loop regions between H1 and H2 (residues 42–58) and between H2 and H3 (residues 71–73), with the remaining residues treated as rigid. DEER distance

constraints were introduced between the nitrogen atoms of the MTSL groups: 42.4 ± 10 Å for C_{628}-C_{425} and 61.7 ± 10 Å for C_{425}-C_{697}.

NMR and PRE Measurements

UBAP1$_C$ peptide was dissolved at a concentration of 1 mg/mL (~0.5 mM) in PBS in D_2O [pD 7.5]. $^1H^{13}C$ gradient-selected HSQC spectra were recorded at natural abundance ^{13}C (1%) at 800 MHz, using a Bruker AVANCE III spectrometer equipped with a TCI (^1H-^{13}C-^{15}N/^2H) cryoprobe with *z* gradients. Pure-shift (PUSH) HSQC spectra (Paudel et al., 2013) were recorded for the uncomplexed peptide. Assignment of the H-C correlations was by HSQC-total correlation spectroscopy and nuclear Overhauser effect spectroscopy spectra, and confirmed by the behavior in the protein complexes. Complexation with HD-PTP$_{CC}$ was detected with the addition of 60 μM of the protein. PRE measurements were recorded by taking MTSL-labeled HD-PTP$_{CC}$ C_{697}S mutant under the same conditions, recording $^1H^{13}C$ HSQCs, and then reducing the MTSL label (rendering it diamagnetic) with 10-fold excess of sodium ascorbate (added from 1 M stock).

Biosensor Binding Studies

Binding studies were performed at 25°C on a multiplex system ProteOn XPR36 (Bio-Rad Laboratories) in 10 mM HEPES (pH 7.4), 150 mM NaCl, 0.05% Tween 20 as running buffer, with 50–100 μg/mL of protein immobilized on an HTE chip (Bio-Rad Laboratories). Peptide solutions (50 μL) were injected at 100 μL/min. Data were analyzed with ProteOn Manager software (Bio-Rad Laboratories), using the equilibrium binding model: Response = $[A] \times R_{max}/([A] + K_D)$ where [A] is the analyte concentration and R_{max} is the maximum response.

Yeast Two-Hybrid Analysis

HD-PTP$_{Bro1\text{-}CC}$, HD-PTP$_{Bro1\text{-}CC}$ F678D, and Alix$_{Bro1\text{-}V}$ cloned into pGBKT7 were used as described previously (Stefani et al., 2011). UBAP1 (122–308) was cloned into pGADT7. Further deletion and missense mutations in UBAP1 as indicated were generated by standard PCR-based mutagenesis. Interactions were tested using the Matchmaker Gold system (Clontech) as described previously (Stefani et al., 2011) Each triplicate experiment was repeated at least three times.

UBAP1 In Vitro Translation and Binding to HD-PTP

UBAP1-strep encoded on a pTriex5 vector (Stefani et al., 2011) was amplified using Pwo Polymerase (Roche). UBAP1 RNA was synthesized from the PCR product using T7 RNA polymerase (Promega). Protein was translated in nuclease-treated rabbit reticulocyte lysate (Promega) containing ^{35}S-methionine (PerkinElmer), and 100 units of RNasin (Promega) for 1 hr at 30°C, followed by 10 min in the presence of 1 mM puromycin. Then 20 μL of translated protein was incubated with 5 μg His$_6$- HD-PTP$_{Bro1\text{-}CC}$ in 250 μL of immunoprecipitation (IP) buffer (20 mM HEPES [pH 7.4], 100 mM NaCl, 1 mM $MgCl_2$, 1% [w/v] Triton X-100) for 2 hr at 4°C, then overnight with 3 μL of anti-His antibody (Clone HIS-1, Sigma). Samples were incubated with 20 μL of protein A-Sepharose beads (Invitrogen) for 2 hr at 4°C, then washed three times in IP buffer.

Mitochondrial Targeting Experiments

Mitochondrial targeting experiments were performed as previously described (Sato et al., 2015). HeLa cells were transiently transfected with HD-PTP$_{Bro1\text{-}CC}$ containing an N-terminal FKBP sequence and a C-terminal myc tag (cloned into pcDNA5), Mito-FRB (a gift from Martin Lowe, Manchester, UK), and either GFP, WT UBAP1-GFP, or F268S UBAP1-GFP (in pEGFP). Mitochondrial relocation of FKBP-HD-PTP$_{Bro1\text{-}CC}$-myc was induced by addition of 1 μM rapamycin (Sigma) for 3 hr. Cells were then prepared for immunofluorescence microscopy as above. Mitochondrial relocation of GFP-tagged constructs was scored in three independent experiments (100 cells counted per experiment).

Cell Culture, Transfection, and siRNA Rescue Experiments

HeLa cells were grown in DMEM with 1% NEAA, 10% fetal calf serum (HyClone; Perbio) and 1% Pen-Strep Fugene 6 (Roche) was used for DNA transfections. Interferin (QBiogene) was used for siRNA, using an HD-PTP nucleotide as previously published (Doyotte et al., 2008), or a non-targeting siRNA (Dharmacon) as a control. Efficient HD-PTP knockdown was confirmed by western blotting, as previously reported (Doyotte et al., 2008). For siRNA rescue experiments, cells were knocked down for 24 hr, then transfected with WT or specified mutants of HD-PTP$_{Bro1\text{-}CC}$ (note that the siRNA oligo targets a C-terminal sequence within HD-PTP) for 48 hr. Rescues were assessed by visual quantification of phenotypes. Cells containing clustered and strongly labeled foci of FK2 staining (previously identified as endosomal; Doyotte et al., 2008) were considered knocked down. Rescued cells displayed a WT, diffuse cytoplasmic distribution of FK2 staining. Scoring was performed for three independent experiments, with at least 100 transfected cells examined each time. Data were subjected to statistical analysis (two-way ANOVA) using Prism5 software (GraphPad). For graphical representation of rescue data, mean scores ± SD are provided for the three determinations.

ACCESSION NUMBERS

The atomic coordinates have been deposited in the PDB (PDB: 5LM1, 5LM2) and resonance assignments have been deposited in the Biological Magnetic Resonance Bank (BMRB: 26914).

SUPPLEMENTAL INFORMATION

Supplemental Information includes five figures and one movie and can be found with this article online at http://dx.doi.org/10.1016/j.str.2016.10.006.

AUTHOR CONTRIBUTIONS

C.L. collected X-ray diffraction data, solved, and refined the crystal structures. D.G. cloned HD-PTP constructs, prepared protein samples, and crystallized the proteins. G.H. prepared spin-labeled proteins and collected DEER data; G.H. and A.J.F. analyzed DEER data. G.H. collected CD data and analyzed it with J.B. M.J.C. collected and analyzed NMR data. P.M. and D.G. collected SPR data and P.M. analyzed it. G.H. and J.B. performed torsion-angle molecular dynamics simulations. F.S. performed the yeast two-hybrid and co-immunoprecipitation experiments. L.W. performed the mitochondrial localization and RNAi rescue experiments. A.J.F. designed and supervised the EPR spectroscopy. P.W. supervised and analyzed all cell biology data. L.T designed and supervised the project and analyzed the structural and biophysical data. L.T and P.W. wrote the paper.

ACKNOWLEDGMENTS

This work was support by the Medical Research Council (MR/K011049/1 to L.T. and P.W.). G.H. is funded by a BBSRC Doctoral Training Studentship. A.J.F. thanks Bruker for sponsorship. All EPR experiments were carried out at the EPSRC National EPR Research Facility & Service. We thank Diamond Light Source for access to beamlines I02 and I03 (MX8997) and their staff for assistance, the staff of the Biomolecular Analysis Facility of the Faculty of Biology Medicine and Health, and the staff at the Advanced Photon Science facilities, University of Manchester. for assistance. We thank Peristera Roboti for help with mitochondrial targeting experiments and Elaine Small for generating FKBP-HD-PTP$_{Bro1\text{-}CC}$-myc. We thank Melanie Vollmar, Simon Tanley and Efrain Ceh Pavia for advice.

Received: July 11, 2016
Revised: September 27, 2016
Accepted: October 12, 2016
Published: November 10, 2016

REFERENCES

Adams, P.D., Afonine, P.V., Bunkoczi, G., Chen, V.B., Davis, I.W., Echols, N., Headd, J.J., Hung, L.W., Kapral, G.J., Grosse-Kunstleve, R.W., et al. (2010). PHENIX: a comprehensive Python-based system for macromolecular structure solution. Acta Crystallogr. D Biol. Crystallogr. *66*, 213–221.

Agromayor, M., Soler, N., Caballe, A., Kueck, T., Freund, S.M., Allen, M.D., Bycroft, M., Perisic, O., Ye, Y., McDonald, B., et al. (2012). The UBAP1 subunit of ESCRT-I interacts with ubiquitin via a SOUBA domain. Structure *20*, 414–428.

Ali, N., Zhang, L., Taylor, S., Mironov, A., Urbe, S., and Woodman, P. (2013). Recruitment of UBPY and ESCRT exchange drive HD-PTP-dependent sorting of EGFR to the MVB. Curr. Biol. *23*, 453–461.

Bissig, C., and Gruenberg, J. (2014). ALIX and the multivesicular endosome: ALIX in Wonderland. Trends Cell Biol. *24*, 19–25.

Brunger, A.T., Adams, P.D., Clore, G.M., DeLano, W.L., Gros, P., Grosse-Kunstleve, R.W., Jiang, J.S., Kuszewski, J., Nilges, M., Pannu, N.S., et al. (1998). Crystallography & NMR system: a new software suite for macromolecular structure determination. Acta Crystallogr. D Biol. Crystallogr. *54*, 905–921.

Carlton, J.G., and Martin-Serrano, J. (2007). Parallels between cytokinesis and retroviral budding: a role for the ESCRT machinery. Science *316*, 1908–1912.

Chen, D.Y., Li, M.Y., Wu, S.Y., Lin, Y.L., Tsai, S.P., Lai, P.L., Lin, Y.T., Kuo, J.C., Meng, T.C., and Chen, G.C. (2012). The Bro1-domain-containing protein Myopic/HDPTP coordinates with Rab4 to regulate cell adhesion and migration. J. Cell Sci. *125*, 4841–4852.

Cheng, Y., Poulos, N.E., Lung, M.L., Hampton, G., Ou, B., Lerman, M.I., and Stanbridge, E.J. (1998). Functional evidence for a nasopharyngeal carcinoma tumor suppressor gene that maps at chromosome 3p21.3. Proc. Natl. Acad. Sci. USA *95*, 3042–3047.

Doyotte, A., Mironov, A., McKenzie, E., and Woodman, P. (2008). The Bro1-related protein HD-PTP/PTPN23 is required for endosomal cargo sorting and multivesicular body morphogenesis. Proc. Natl. Acad. Sci. USA *105*, 6308–6313.

Emsley, P., Lohkamp, B., Scott, W.G., and Cowtan, K. (2010). Features and development of Coot. Acta Crystallogr. D Biol. Crystallogr. *66*, 486–501.

Fisher, R.D., Chung, H.Y., Zhai, Q., Robinson, H., Sundquist, W.I., and Hill, C.P. (2007). Structural and biochemical studies of ALIX/AIP1 and its role in retrovirus budding. Cell *128*, 841–852.

Hurley, J.H. (2015). ESCRTs are everywhere. EMBO J. *34*, 2398–2407.

Ichioka, F., Takaya, E., Suzuki, H., Kajigaya, S., Buchman, V.L., Shibata, H., and Maki, M. (2007). HD-PTP and Alix share some membrane-traffic related proteins that interact with their Bro1 domains or proline-rich regions. Arch. Biochem. Biophys. *457*, 142–149.

Jeschke, G., Chechik, V., Ionita, P., Godt, A., Zimmermann, H., Banham, J., Timmel, C.R., Hilger, D., and Jung, H. (2006). DeerAnalysis2006-a comprehensive software package for analyzing pulsed ELDOR data. Appl. Magn. Reson. *30*, 473–498.

Kabsch, W. (2010). XDS. Acta Crystallogr. D Biol. Crystallogr. *66*, 125–132.

Kharitidi, D., Apaja, P.M., Manteghi, S., Suzuki, K., Malitskaya, E., Roldan, A., Gingras, M.C., Takagi, J., Lukacs, G.L., and Pause, A. (2015). Interplay of endosomal pH and ligand occupancy in integrin alpha5beta1 ubiquitination, endocytic sorting, and cell migration. Cell Rep. *13*, 599–609.

Kim, J., Sitaraman, S., Hierro, A., Beach, B.M., Odorizzi, G., and Hurley, J.H. (2005). Structural basis for endosomal targeting by the Bro1 domain. Dev. Cell *8*, 937–947.

Kimura, Y., Tanigawa, M., Kawawaki, J., Takagi, K., Mizushima, T., Maeda, T., and Tanaka, K. (2015). Conserved mode of interaction between yeast Bro1 family V domains and YP(X)nL motif-containing target proteins. Eukaryot. Cell *14*, 976–982.

Kok, K., Naylor, S.L., and Buys, C.H. (1997). Deletions of the short arm of chromosome 3 in solid tumors and the search for suppressor genes. Adv. Cancer Res. *71*, 27–92.

Lee, S., Joshi, A., Nagashima, K., Freed, E.O., and Hurley, J.H. (2007). Structural basis for viral late-domain binding to Alix. Nat. Struct. Mol. Biol. *14*, 194–199.

Lee, J., Oh, K.J., Lee, D., Kim, B.Y., Choi, J.S., Ku, B., and Kim, S.J. (2016). Structural study of the HD-PTP Bro1 domain in a complex with the core region of STAM2, a subunit of ESCRT-0. PLoS One *11*, e0149113.

Lin, G., Aranda, V., Muthuswamy, S.K., and Tonks, N.K. (2011). Identification of PTPN23 as a novel regulator of cell invasion in mammary epithelial cells from a loss-of-function screen of the 'PTP-ome'. Genes Dev. *25*, 1412–1425.

Ma, H., Wardega, P., Mazaud, D., Klosowska-Wardega, A., Jurek, A., Engstrom, U., Lennartsson, J., and Heldin, C.H. (2015). Histidine-domain-containing protein tyrosine phosphatase regulates platelet-derived growth factor receptor intracellular sorting and degradation. Cell Signal. *27*, 2209–2219.

Manteghi, S., Gingras, M.C., Kharitidi, D., Galarneau, L., Marques, M., Yan, M., Cencic, R., Robert, F., Paquet, M., Witcher, M., et al. (2016). Haploinsufficiency of the ESCRT component HD-PTP predisposes to Cancer. Cell Rep. *15*, 1893–1900.

Mariotti, M., Castiglioni, S., Garcia-Manteiga, J.M., Beguinot, L., and Maier, J.A. (2009). HD-PTP inhibits endothelial migration through its interaction with Src. Int. J. Biochem. Cell Biol. *41*, 687–693.

Morita, E., Sandrin, V., Chung, H.Y., Morham, S.G., Gygi, S.P., Rodesch, C.K., and Sundquist, W.I. (2007). Human ESCRT and ALIX proteins interact with proteins of the midbody and function in cytokinesis. EMBO J. *26*, 4215–4227.

Parkinson, M.D., Piper, S.C., Bright, N.A., Evans, J.L., Boname, J.M., Bowers, K., Lehner, P.J., and Luzio, J.P. (2015). A non-canonical ESCRT pathway, including histidine domain phosphotyrosine phosphatase (HD-PTP), is used for down-regulation of virally ubiquitinated MHC class I. Biochem. J. *471*, 79–88.

Pashkova, N., Gakhar, L., Winistorfer, S.C., Sunshine, A.B., Rich, M., Dunham, M.J., Yu, L., and Piper, R.C. (2013). The yeast Alix homolog Bro1 functions as a ubiquitin receptor for protein sorting into multivesicular endosomes. Dev. Cell *25*, 520–533.

Paudel, L., Adams, R.W., Kiraly, P., Aguilar, J.A., Foroozandeh, M., Cliff, M.J., Nilsson, M., Sandor, P., Waltho, J.P., and Morris, G.A. (2013). Simultaneously enhancing spectral resolution and sensitivity in heteronuclear correlation NMR spectroscopy. Angew. Chem. *52*, 11616–11619.

Pires, R., Hartlieb, B., Signor, L., Schoehn, G., Lata, S., Roessle, M., Moriscot, C., Popov, S., Hinz, A., Jamin, M., et al. (2009). A crescent-shaped ALIX dimer targets ESCRT-III CHMP4 filaments. Structure *17*, 843–856.

Polyhach, Y., Bordignon, E., and Jeschke, G. (2011). Rotamer libraries of spin labelled cysteines for protein studies. Phys. Chem. Chem. Phys. *13*, 2356–2366.

Qian, J., Yang, J., Zhang, X., Zhang, B., Wang, J., Zhou, M., Tang, K., Li, W., Zeng, Z., Zhao, X., et al. (2001). Isolation and characterization of a novel cDNA, UBAP1, derived from the tumor suppressor locus in human chromosome 9p21-22. J. Cancer Res. Clin. Oncol. *127*, 613–618.

Rollinson, S., Rizzu, P., Sikkink, S., Baker, M., Halliwell, N., Snowden, J., Traynor, B.J., Ruano, D., Cairns, N., Rohrer, J.D., et al. (2009). Ubiquitin associated protein 1 is a risk factor for frontotemporal lobar degeneration. Neurobiol. Aging *30*, 656–665.

Sato, K., Roboti, P., Mironov, A.A., and Lowe, M. (2015). Coupling of vesicle tethering and Rab binding is required for in vivo functionality of the golgin GMAP-210. Mol. Biol. Cell *26*, 537–553.

Schmidt, M.H., Hoeller, D., Yu, J., Furnari, F.B., Cavenee, W.K., Dikic, I., and Bogler, O. (2004). Alix/AIP1 antagonizes epidermal growth factor receptor downregulation by the Cbl-SETA/CIN85 complex. Mol. Cell. Biol. *24*, 8981–8993.

Sette, P., Mu, R., Dussupt, V., Jiang, J., Snyder, G., Smith, P., Xiao, T.S., and Bouamr, F. (2011). The Phe105 loop of Alix Bro1 domain plays a key role in HIV-1 release. Structure *19*, 1485–1495.

Stefani, F., Zhang, L., Taylor, S., Donovan, J., Rollinson, S., Doyotte, A., Brownhill, K., Bennion, J., Pickering-Brown, S., and Woodman, P. (2011). UBAP1 is a component of an endosome-specific ESCRT-I complex that is essential for MVB sorting. Curr. Biol. *21*, 1245–1250.

Sun, S., Zhou, X., Zhang, W., Gallick, G.E., and Kuang, J. (2015). Unravelling the pivotal role of Alix in MVB sorting and silencing of the activated EGFR. Biochem. J. *466*, 475–487.

Toyooka, S., Ouchida, M., Jitsumori, Y., Tsukuda, K., Sakai, A., Nakamura, A., Shimizu, N., and Shimizu, K. (2000). HD-PTP: a novel protein tyrosine phosphatase gene on human chromosome 3p21.3. Biochem. Biophys. Res. Commun. *278*, 671–678.

von Schwedler, U.K., Stuchell, M., Muller, B., Ward, D.M., Chung, H.Y., Morita, E., Wang, H.E., Davis, T., He, G.P., Cimbora, D.M., et al. (2003). The protein network of HIV budding. Cell *114*, 701–713.

Walshaw, J., and Woolfson, D.N. (2001). Socket: a program for identifying and analysing coiled-coil motifs within protein structures. J. Mol. Biol. *307*, 1427–1450.

Wunderley, L., Brownhill, K., Stefani, F., Tabernero, L., and Woodman, P. (2014). The molecular basis for selective assembly of the UBAP1-containing endosome-specific ESCRT-I complex. J. Cell Sci. *127*, 663–672.

Zhai, Q., Fisher, R.D., Chung, H.Y., Myszka, D.G., Sundquist, W.I., and Hill, C.P. (2008). Structural and functional studies of ALIX interactions with YPX(n)L late domains of HIV-1 and EIAV. Nat. Struct. Mol. Biol. *15*, 43–49.

Zhai, Q., Landesman, M.B., Robinson, H., Sundquist, W.I., and Hill, C.P. (2011). Identification and structural characterization of the ALIX-binding late domains of simian immunodeficiency virus SIVmac239 and SIVagmTan-1. J. Virol. *85*, 632–637.

Structure, Volume *24*

Supplemental Information

Structural Basis for Selective Interaction between the ESCRT Regulator HD-PTP and UBAP1

Deepankar Gahloth, Colin Levy, Graham Heaven, Flavia Stefani, Lydia Wunderley, Paul Mould, Matthew J. Cliff, Jordi Bella, Alistair J. Fielding, Philip Woodman, and Lydia Tabernero

Supplementary Information

Supplementary Figures: S1, S2, S3, S4 and S5

Supplementary Movie 1

Supplementary Figure S1

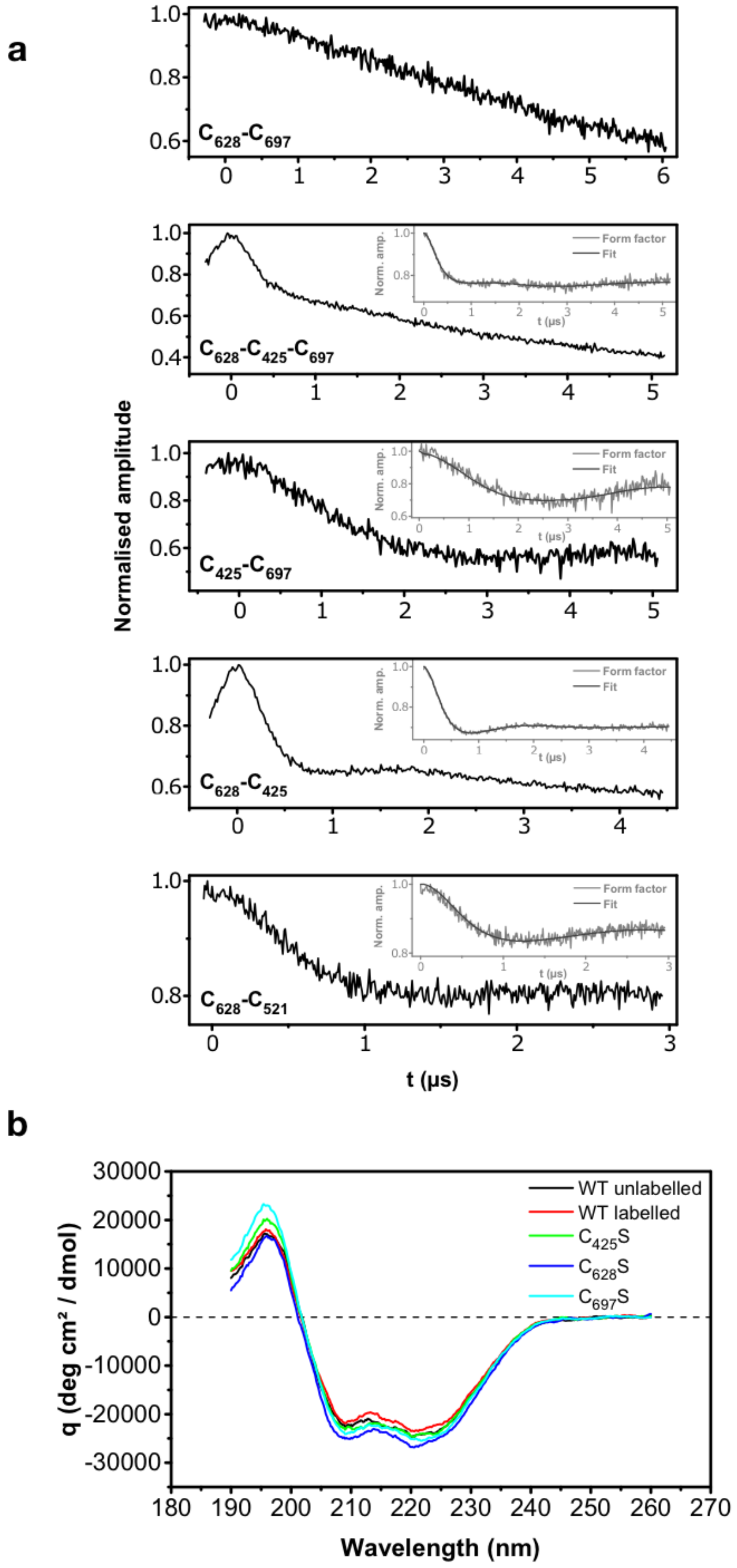

Figure S1. **(Related to Figure 2) DEER spectroscopy of HD-PTP$_{CC}$ and Circular Dichrosim analysis of MTSL-labelled HD-PTP$_{CC}$ and mutants used in the EPR studies.**

(a) DEER traces of the triply MTSL-labelled HD-PTP$_{CC}$ and doubly MTSL-labelled mutants, as indicated inside each frame. Inset graphs show form factor fits after exponential background correction. Modulation is apparent in all triply and doubly labelled proteins except for the C_{425}S mutant (top panel) where the distance between spins is larger than the detectable limits within this window size. **(b)** Circular dichroism of HD-PTP$_{CC}$ (unlabelled and triply labelled at C_{425}, C_{628} and C_{697}) and mutants C_{425}S (labelled at C_{628} and C_{697}), C_{628}S (labelled at C_{425} and C_{697}) and C_{697}S (labelled at C_{425} and C_{628}). Samples for circular dichroism were adjusted to 0.1 mg/mL and buffer exchanged into phosphate buffer (100 mM potassium phosphate, 100 mM potassium fluoride, pH 7.4). Spectra were recorded on a Jasco J-180 spectropolarimeter between 190 and 260 nm using cuvettes of 0.5 mm path length. A data pitch of 0.2 nm was used with a response time of 8 seconds per point. Circular dichroism mdeg units were converted to mean residue ellipticity. No significant changes are seen upon labelling or mutagenesis of HD-PTP$_{CC}$, thus confirming that there are no alterations to the secondary structure.

Supplementary Figure S2

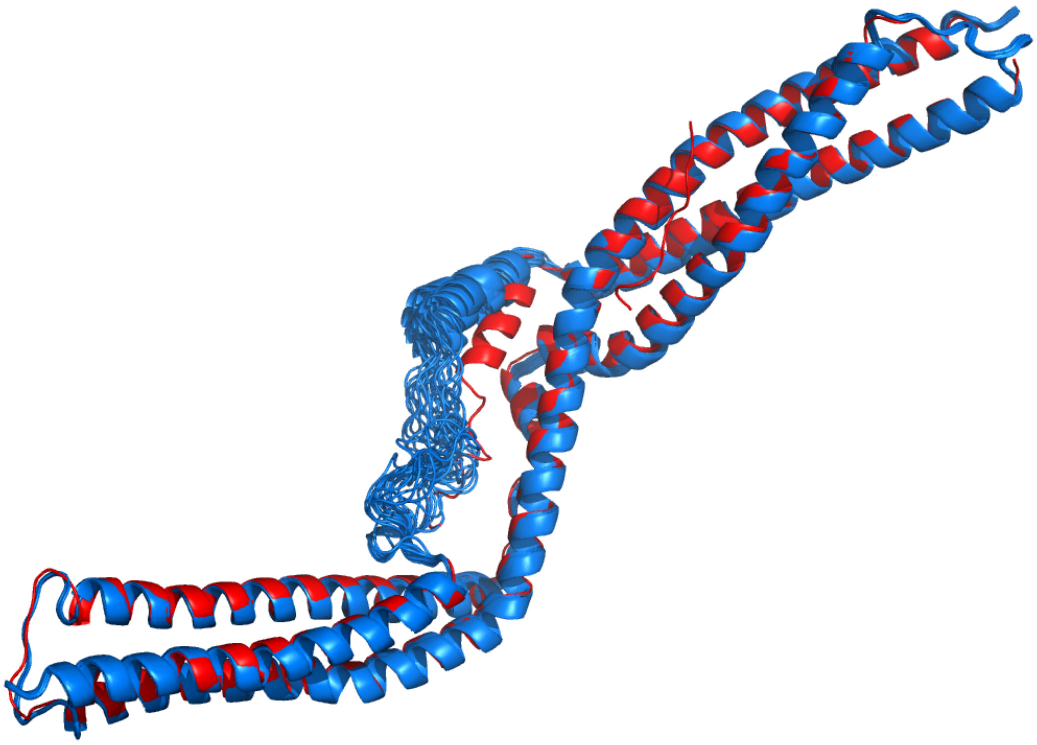

Figure S2. (Related to Figure 2) Torsion-angle molecular dynamics simulations using DEER distance constraints.

Ribbon diagram of the $HDPTP_{CC}$ crystal structure (red) superimposed to 25 trajectory model structures at 1 ns intervals (blue) showing the flexibility of the loops that connect helix H2 with the rest of the CC domain. Several orientations of helix H2 resulting from the molecular dynamics simulations are compatible with the interspin distances estimated form the DEER experiments, without major global conformational changes of the CC domain structure.

Supplementary Figure S3

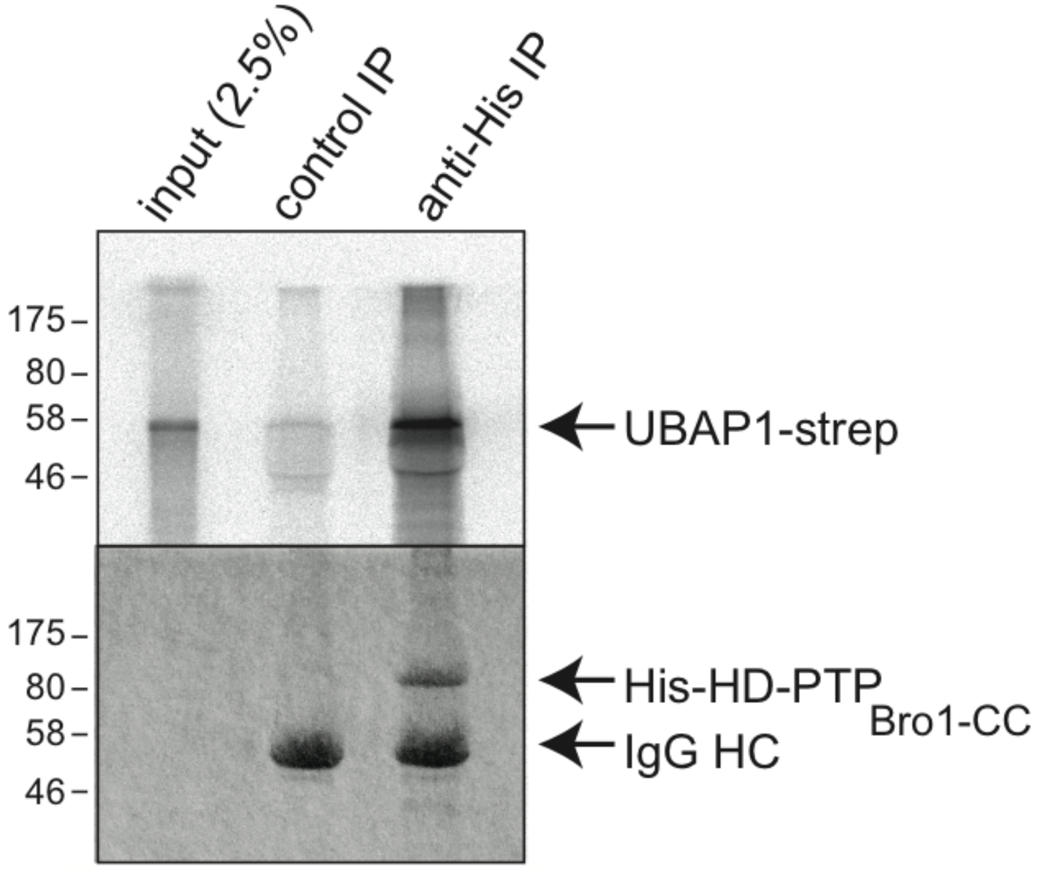

Figure S3. (Related to Figure 3) Co-immunoprecipitation of UBAP1 with HD-PTP.

UBAP1-strep was translated *in vitro* and incubated with or without His_6-HD-$PTP_{Bro1\text{-}CC}$. Samples were immunoprecipitated with anti-His antobodies. Top panel: phosphorimage. Bottom panel: Coomassie stained SDS-PAGE.

Supplementary Figure S4

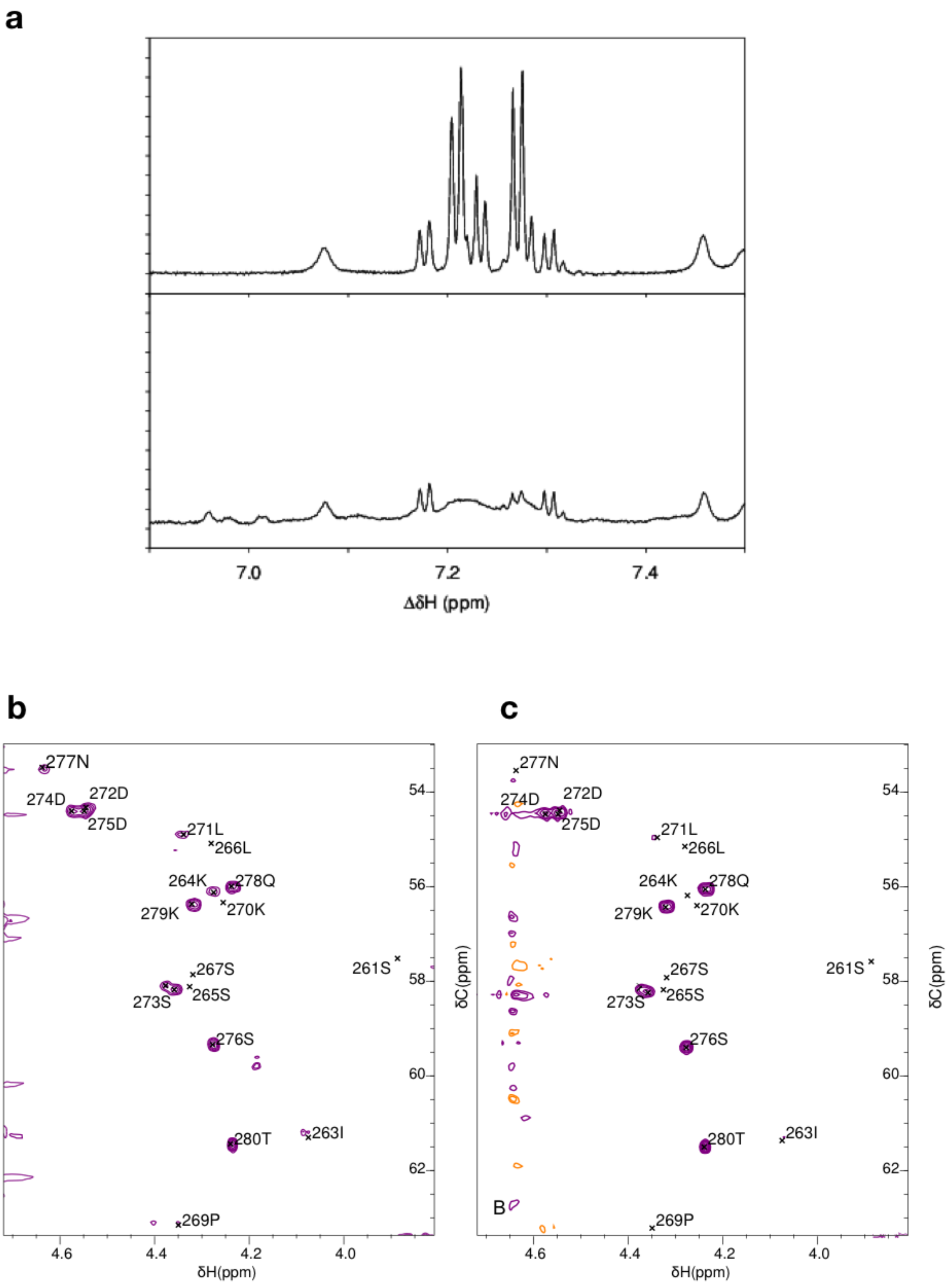

Figure S4. (Related to Figure 5) NMR and PRE studies of UBAP1 peptide in the presence of HD-PTP.

(a) Aromatic regions of ^{1}H NMR spectra of UBAP1$_{C}$, in the absence (upper panel) and presence (lower panel) of HD-PTP$_{CC}$. Narrow line-width signals arising from the sidechain of Phe268 (Hδ# (7.21 ppm), Hε# (7.28 ppm) and Hζ (7.24 ppm)) are clearly visible in the top panel, but absent from the lower one. Present at approximately 25% intensity are signals (7.18, 7.29 and 7.30 ppm) arising from UBAP1 F268 in the *cis* proline isomer of the peptide, which are unaffected by the presence of HD-PTP$_{CC}$. **(b-c)** Effect of electron spin-label at C$_{425}$: Hα region of ^{1}H-^{13}C HSQC of UBAP1$_{C}$ in the presence of spin-labelled variant of HD-PTP$_{CC}$ (10:1 mixture UBAP1$_{C}$:HD-PTP$_{CC}$); **(b)** shows spectrum when spin-label is diamagnetic (reduced with excess ascorbate), and **(c)** shows spectrum when spin-label is paramagnetic.

Supplementary Figure S5

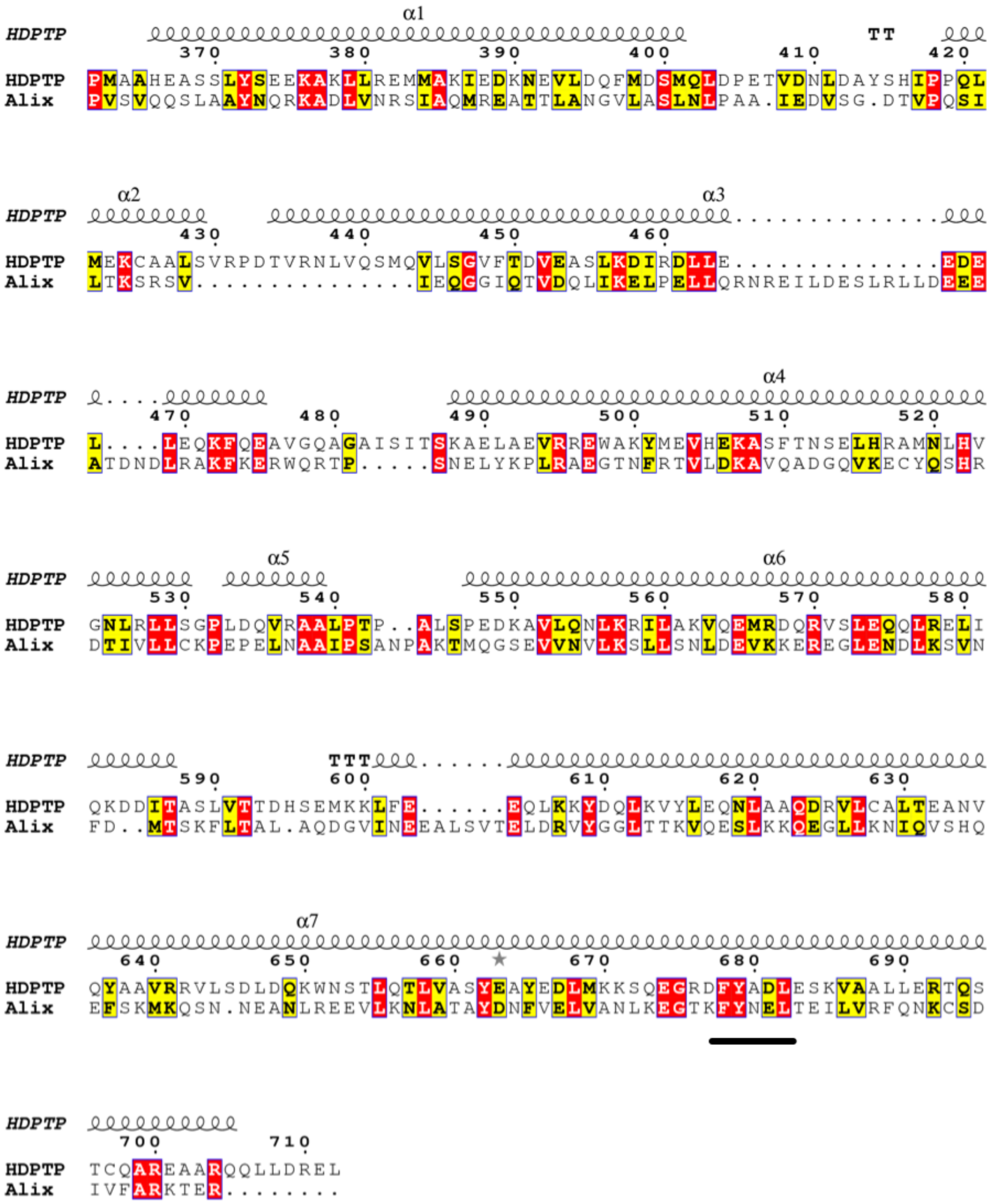

Figure S5. **(Related to Figure 6) Sequence alignment of the coiled-coil domains of human HD-PTP and Alix.**

Alignment of the sequences of the coiled-coil domain for HD-PTP and Alix. Conservation is highlighted in yellow and identity in red. The conserved FYX_nL motif is underlined in black. Alignment was done using ESPript 3 online server (Robert and Gouet, 2014).

Movie 1. (Related to Figure 4)

A cartoon representation of the coiled coil domain of HD-PTP (HD-PTP_{CC}) phosphatase in complex with UBAP1. HD-PTP_{CC} is shown in surface representation and coloured by electrostatic potential. The UBAP1 peptide is shown in stick representation and coloured by atom type. Two electron density maps are displayed for the peptide, an omit map contoured at 2.5σ (green) and a 1σ feature enhanced map (blue) (Afonine et al., 2015). This movie was generated in CCP4MG (McNicholas et al., 2011)

References

Afonine, P. V., Moriarty, N. W., Mustyakimov, M., Sobolev, O. V., Terwilliger, T. C., Turk, D., Urzhumtsev, A. & Adams, P. D. 2015. Fem: Feature-Enhanced Map. *Acta Crystallographica Section D-Structural Biology,* 71**,** 646-666.

Mcnicholas, S., Potterton, E., Wilson, K. S. & Noble, M. E. 2011. Presenting Your Structures: The Ccp4mg Molecular-Graphics Software. *Acta Crystallogr D Biol Crystallogr,* 67**,** 386-94.

Robert, X. & Gouet, P. 2014. Deciphering Key Features In Protein Structures With The New Endscript Server. *Nucleic Acids Res,* 42**,** W320-4.
